# Critical Appraisal of Pharmaceutical Therapy in Diabetic Cardiomyopathy—Challenges and Prospectives

**DOI:** 10.3390/ph18010134

**Published:** 2025-01-20

**Authors:** Elina Khattab, Michaelia Kyriakou, Elena Leonidou, Stefanos Sokratous, Angeliki Mouzarou, Michael M. Myrianthefs, Nikolaos P. E. Kadoglou

**Affiliations:** 1 Department of Cardiology, Nicosia General Hospital, 2029 Nicosia, Cyprus; khattab_elina@outlook.com (E.K.); michaelakyriakou7@gmail.com (M.K.); stefanossokratous94@gmail.com (S.S.); myr.michael@shso.org.cy (M.M.M.); 2 Department of Cardiology, Limassol General Hospital, 3304 Limassol, Cyprus; leonidouelena94@hotmail.co.uk; 3 Department of Cardiology, Pafos General Hospital, 8026 Paphos, Cyprus; mouzarou@gmail.com; 4 Medical School, University of Cyprus, 1678 Nicosia, Cyprus

**Keywords:** diabetic cardiomyopathy, heart failure, ARNIs, SGLT2i, pharmaceutical therapy

## Abstract

Diabetes mellitus (DM) is a multifaceted disorder with a pandemic spread and a remarkable burden of cardiovascular mortality and morbidity. Diabetic cardiomyopathy (DBCM) has been increasingly recognized as the development of cardiac dysfunction, which is accompanied by heart failure (HF) symptoms in the absence of obvious reasons like ischemic heart disease, hypertension, or valvulopathies. Several pathophysiological mechanisms have been proposed, including metabolic disorders (e.g., glycation products), oxidative stress, low-grade inflammation, mitochondrial dysfunction, etc., which should guide the development of new therapeutic strategies. Up to now, HF treatment has not differed between patients with and without diabetes, which limits the expected benefits despite the high cardiovascular risk in the former group. However, DBCM patients may require different management, which prioritize anti-diabetic medications or testing other novel therapies. This review aims to appraise the challenges and prospectives of the individualized pharmaceutical therapy for DBCM.

## 1. Introduction

Diabetic cardiomyopathy (DBCM) has been increasingly recognized as the development of cardiac dysfunction, which is accompanied by symptoms of heart failure (HF) in the absence of obvious underlying reasons like myocardial ischemia, hypertension, or valvulopathies [1]. DBCM represents a unique pathological entity with a specific set of mechanisms driving cardiac dysfunction, which underlines the importance of targeted research to elucidate its pathophysiology and develop precise pharmaceutical interventions. Its management usually falls within the context of HF therapeutic algorithms, but its distinct pathophysiology and significant cautions in the diabetic population should be taken into consideration to balance potential harms and long-term prognostic benefits. Unfortunately, the literature focusing entirely on DBCM is limited.

The first small part of our review aims to link pathophysiology with clinical phenotypes. From the pathological perspective, structural changes are characterized by left ventricle (LV) stiffening and remodeling, which are related to fibrosis and parenchymal fatty dystrophy of the myocardium [2]. These changes are merely secondary to diabetes-associated complications, but they are predominantly attributed to the molecular and cellular derangements of DBCM itself. The underlying pathophysiology is quite complex and includes an interplay between hyperglycemia, oxidative stress, inflammation, microvascular dysfunction, neurohormonal abnormalities, and lipotoxicity; however it remains elusive [3]. In the present review, we have gathered and presented the most important and well-documented pathophysiological mechanisms. Apart from neurohormonal abnormalities, the reader can easily recognize the involvement of unique diabetes-related pathophysiology.

The second bigger part of our review focuses on pharmaceutical therapy. The recommended anti-diabetic medications remain the cornerstone for DBCM prevention and could contribute to the essential therapy of patients with established DBCM. Among a long list of available hypoglycemic agents, metformin, sodium–glucose cotransporter-2 inhibitors (SGLT-2i), and Dual GIP/GLP-1 Receptor Agonists have been associated with beneficial effects on cardiac remodeling and function [4,5,6]. Combinations of anti-diabetic medications may be even more beneficial than using each single agent at maximum dose. Over and above that, systematic exercise should always be considered as an adjunct therapy for DBCM [7]. Regarding classical HF therapy, this should be applied to patients with DBCM and reduced ejection fraction (HFrEF) to improve symptoms and prognosis. It should be clarified whether conventional HF therapies are equivalently effective in cohorts with well-defined DBCM since there are significant cautions (e.g., diabetes exacerbation with diuretics) and different pathophysiological mechanisms. Notably, a significant proportion of DBCM patients present heart failure with preserved ejection fraction (HFpEF) phenotype [5]. In the latter case, classical HF therapy is capable of relieving symptoms, but only the trials using SGLT-2i have shown robust evidence for their beneficial effects on prognosis, compared to other medications [8]. Emerging therapies, including natural products, microRNAs, etc., could fill the gap in pharmaceutical therapy for patients with DBCM and HFpEF phenotype.

The present review provides a critical appraisal of the currently available pharmaceutical therapy for DBCM, shedding light on challenges and perspectives. To optimize anti-HF therapy for DBCM, it is essential to understand the pathogenesis, explore the progression, and monitor the long-term impact on cardiac function. Overall, the potential interplay between various mechanisms, most of which not well-known, makes the therapy of DBCM challenging. It also questions the currently recommended pharmaceutical therapy since it is oriented to the traditional pathophysiological mechanisms of HF, like renin–angiotensin–aldosterone system (RAAS) and sympathetic system overactivation, while there are additional important mechanisms as described in the following paragraphs.

## 2. Pathophysiology of Diabetic Cardiomyopathy

The pathophysiology of DBCM is highly complex and multifactorial, predominantly driven by insulin resistance (in type 2 diabetes mellitus-T2DM) and chronic hyperglycemia (in both types of diabetes). Oxidative stress and chronic low-grade inflammation have been recognized as important causative factors of DBCM as well. Recently, a growing number of other pathways have been proposed as contributors to the gradual impairment of cardiac function in the diabetic state [1]. Unraveling the pathophysiology of DBCM will open a new window to novel tailored therapies with higher efficacy.

### 2.1. Hyperglycemia, Oxidative Stress and Inflammation

In DBCM, there are several pathways of oxidative stress exacerbation: (1) Chronic hyperglycemia enhances the utilization of Nicotinamide Adenine Dinucleotide Phosphate Hydrogen (NADPH) in the oxidative-prone polyol pathway and induces the intracellular deposit of advanced glycation end-products (AGEs) and the expression of their receptors (RAGEs). Their cross-linking with collagen and other extracellular matrix components results in increased myocardial stiffness and diastolic dysfunction [9]. (2) In addition to AGE formation, hyperglycemia inhibits nuclear factor erythroid 2-related factor 2 (Nrf2) and Sirtuin 1 (Sirt1)-mediated antioxidant signaling and activates NF-κB-mediated inflammatory pathways, creating a vicious cycle of oxidative stress and inflammation interaction causing further damage of the cardiac muscle [3,10]. Thus, it is extremely important to disrupt this detrimental cycle by pharmaceutical therapy. Therapies like nucleotide-binding oligomerization, pyrin domain-containing 3 (NLRP3) inflammasome inhibitors, and antioxidants directly address these pathways with the potential to alleviate myocardial damage. (3) The diversion of mitochondrial glucose oxidation to free-fatty acid (FFA) oxidation increases free electron formation, resulting in mitochondrial proton leak and accumulation of reactive oxygen species (ROS). Progressive mitochondrial damage leads to lipid accumulation and further exaggeration of ROS production, thereby triggering the production of oxidative stress derivatives and AGEs [11]. (4) Additionally, hyperinsulinemia induces phosphorylation of MAPK, a pathway with multifaced effects in cardiac hypertrophy regulation, TNF-α and IL-6 release, NLRP3 inflammasome, and other metabolic derangements [12,13]. Novel agents targeting MAPK pathways, such as curcumin, can potentially reduce inflammation and fibrosis in DBCM, but these preclinical data require clinical evaluation [14].

### 2.2. Lipotoxicity

In DM, cardiac glucose uptake and glycolysis are diminished due to suppressed AMP-activated protein kinase (AMPK) activation and insulin resistance. There is a metabolic shift from glucose to FFA utilization from cardiomyocytes [15,16]. This is less efficient, thus imposing energy deficiency and additional strain on the myocardium. Those effects, combined with high oxidative stress, led to a lipotoxic cardiac injury characterized by the accumulation of triglycerides and toxic lipid intermediates such as diacylglycerol and ceramides within cardiomyocytes. All these changes end in cellular damage, cardiomyocyte apoptosis, myocardial fibrosis, and contractile dysfunction [17]. Lipotoxicity significantly contributes to cardiac dysfunction, and novel therapies, including flavonoids, aim to mitigate these effects by enhancing AMPK activity and reducing lipid accumulation [18].

### 2.3. Microvascular Dysfunction

It is increasingly recognized as a causative factor of DBCM, characterized by impaired microvascular dilatation due to decreased eNOS expression, reduced nitric oxide (NO) production, and endothelial impairment. The underlying mechanisms include hyperglycemia-induced protein kinase C (PKC) signaling and AGE formation [19], insulin resistance-related disruption of phosphoinositide 3-kinase/Akt PI3K/Akt signaling, and elevated ROS in cardiac cells [20]. Preliminary data derived from interventions targeting endothelial dysfunction, such as antioxidants and aldose reductase inhibitors, offer promising avenues to restore vascular health and reduce cardiac complications [21]. Unfortunately, current clinical data are missing.

### 2.4. Neurohormonal Abnormalities

Overactivation of the RAAS in DM leads to excessive angiotensin II production, driving cardiac fibrosis via increased collagen synthesis, cardiomyocyte apoptosis, vascular inflammation, and oxidative stress [22]. Sympathetic overactivation is common in chronic DM, causing myocardial fibrosis and abnormal β-adrenergic signaling, reduced myocardial contractility, and impaired cardiac kinetics [23]. Also, aldosterone induces cardiac fibrosis through several mechanisms, including the stimulation of ROS formation via NADPH oxidase activation, mitochondrial dysfunction, and nuclear factor kappa B (NFκB) signaling, which promote inflammation and the expression of profibrotic molecules such as transforming growth factor-beta 1 (TGF-β1), plasminogen activator inhibitor 1 (PAI-1), and endothelin-1 [24]. Targeting RAAS overactivation has long been associated with significant improvement in cardiac remodeling, cardiac function, and prognosis in HFrEF patients. Most recently, mitochondrial antioxidants and other agents provide the potential to disrupt the RAAS profibrotic pathway, which could ameliorate cardiac function in DBCM [21].

### 2.5. Autophagy

It is a normal process of homeostasis, which degrades and recycles cellular components. Dysregulated autophagy can lead to cardiomyocyte damage and death, contributing to the progression of DBCM. Several key molecular pathways are involved in the regulation of autophagy in DBCM, including AMPK, mTOR, FOXOs, SIRTs, and the PINK1/Parkin pathway [25]. Excessive mitophagy, a type of autophagy that occurs in dysfunctional mitochondria, is observed in diabetes and may further exacerbate myocardial damage [26].

### 2.6. Calcium Homeostasis

It is an integral part of cardiac function, regulating heart muscle contraction and relaxation. In DBCM, there is an imbalance since sarco/endoplasmic reticulum Ca^2+^-ATPase, SERCA, and ryanodine receptors (RyR2) are compromised, hindering calcium reuptake into the sarcoplasmic reticulum and erratic calcium release [27]. Dysregulation of Ca homeostasis exacerbates both diastolic and systolic function and is pro-arrhythmogenic [28].

### 2.7. MicroRNAs (miRNAs)

Small, single-stranded, non-coding RNAs act as posttranscriptional regulators of gene expression by binding to the 3′ untranslated region (3′-UTR) of target mRNA, leading to mRNA degradation or translational repression [29]. Although the precise mechanisms by which subcellular miRNAs influence DBCM remain unclear, there is growing evidence from animal models suggesting a significant correlation between their dysregulation and DBCM development [30]. However, due to word count constraints, a more comprehensive exploration of this topic will be addressed in future dedicated reviews.

## 3. Clinical Phenotypes

**Figure 1:**
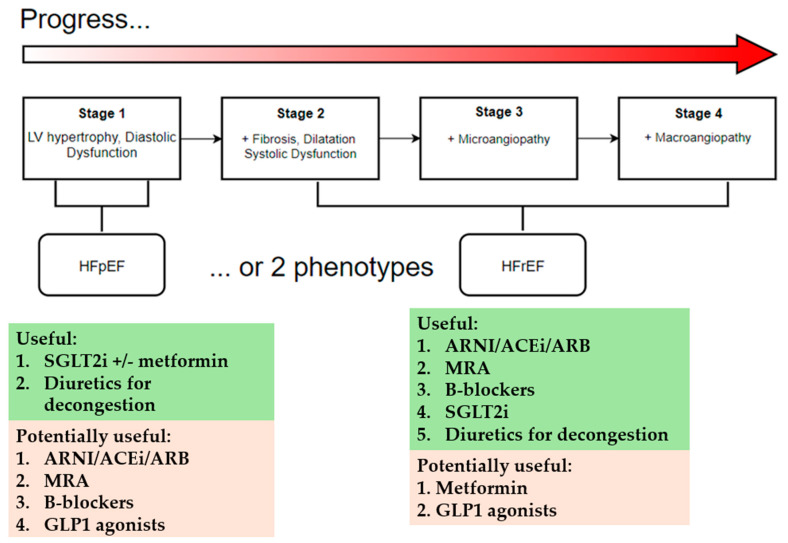
Phenotypes of DBCM and the recommended heart failure therapy.

Two prevailing theories on DBCM evolution exist: one posits a single phenotype transitioning from diastolic to systolic dysfunction [31], while the other suggests two distinct phenotypes: (A) Systolic dysfunction leading to dilated LV and HFrEF; (B) Diastolic dysfunction with more restrictive pattern and HFpEF [32,33]. Figure 1 illustrates both theories, suggesting that DBCM can be a progressive disease with gradual specific structural and functional abnormalities. At early stages, DBCM patients may be asymptomatic with marked LV hypertrophy (LVH) and diastolic dysfunction, while later stages are characterized by fibrosis, ventricular stiffening and dilatation, and systolic dysfunction, leading to overt HFrEF [34].

### Obesity and Cardiomyopathy

Obesity is commonly observed in patients with DBCM and is one of the comorbidities associated with HFpEF. In such patients, increased levels of systemic pro-inflammatory cytokines, fatty acids, and glucose, along with impaired angiogenesis, contribute to pathological hypertrophy and diastolic dysfunction [35]. In the long-term, obesity contributes to cardiac remodeling, which is characterized by left ventricular hypertrophy, cardiac fibrosis, and diastolic dysfunction, which can progress to overt HFpEF [36]. Shared mechanisms, including lipotoxicity, oxidative stress, and systemic inflammation, play a crucial role in both obesity and DBcM, underscoring the importance of therapeutic approaches that target these pathways to improve treatment outcomes [35]. It remains to be proven whether novel agents targeting weight loss could, in parallel, halt the development and progression of DBCM.

## 4. Classical Anti-Diabetic Therapy

Traditional treatments for DM are mainly focused on controlling blood glucose levels, but they may also contribute to the prevention of DBCM and HF by improving cardiac function and positively impacting clinical outcomes [37]. However, certain anti-diabetic medications have been associated with worsening cardiac function, HF hospitalizations (e.g., glitazones), and other adverse cardiovascular issues (e.g., sulfonylureas). Following these negative effects, the priority in clinical trials evaluating anti-diabetic drugs has been the cardiovascular safety of testes agents before their use in clinical practice. While HF treatment has not been distinguished between patients with and without diabetes, the effects of anti-diabetic medications on HF can differ (Table 1). This emphasizes the importance of prioritizing drugs that are safe and effective in slowing the progression of DBCM, improving cardiac function, and reducing HF-related events [5,38].

### 4.1. Metformin

Metformin, an old, effective, and cheap antidiabetic medication, has been the first-choice treatment for patients with type 2 diabetes since 2005. It mainly reduces the production of glucose in the liver and improves insulin sensitivity [39]. In addition to glucose-lowering effects, it plays a crucial role in the prevention of DBCM and HF. According to the recent ESC guidelines for the diagnosis and treatment of acute and chronic HF, metformin is considered safe for use in patients with HF. This recommendation derives from observational studies, which suggests a preference for metformin over insulin and sulfonylureas in T2DM [5,38,40]. Metformin activates AMPK, which improves myocardial energy balance and reduces oxidative stress and inflammation—key factors in the pathogenesis of DBCM [41,42]. Most importantly, clinical studies have demonstrated reduced incidence of HF and cardiovascular events, particularly in patients with established heart disease [37,38]. In terms of cardiac function, a small retrospective cohort study in 2010 involving 242 subjects suggested that metformin may improve LV—diastolic function in patients with T2DM, HF, or coronary artery disease (CAD) [4]. Metformin may also improve LV function, reduce LV mass, and enhance diastolic function, leading to better myocardial performance and less inflammatory burden [43,44,45,46,47,48]. Additionally, cardiac magnetic resonance (CMR) studies have indicated that metformin decreases myocardial steatosis and fibrosis, associated with better cardiac outcomes [49].

The combination of metformin and SGLT2 inhibitors has emerged as a superior therapeutic approach compared to monotherapy with either agent alone, establishing it as a first-line therapy for diabetic patients with heart disease and eventually of DBCM [50]. Metformin primarily improves insulin sensitivity and suppresses hepatic glucose production, while SGLT2 inhibitors enhance urinary glucose excretion and confer significant cardiovascular benefits [51]. By addressing distinct yet complementary mechanisms of action, this combination effectively targets key pathophysiological pathways in DBCM, including glycemic control, insulin resistance, and cardiovascular remodeling, providing a more holistic treatment strategy. Large clinical trials remain to prove the cardiac efficacy of this combination in patients with clearly defined DBCM.

### 4.2. Insulin

Insulin, discovered in 1921, is critical for managing hyperglycemia, particularly in T1DM and advanced T2DM [52]. While effective in controlling blood sugar, its role in preventing DBCM is complex. Insulin can lead to hyperinsulinemia, raising concerns about fluid retention in HF patients due to increased sodium retention in the kidneys. This may negatively impact cardiac function and can accelerate HF progression, as shown in observational studies like CHARM [6,53]. Despite these risks, tight glycemic control with insulin, especially early in the disease, can reduce the risk of DBCM. Insulin also has direct effects on the heart, influencing glucose uptake in myocardial cells, cardiac contractility, and vascular function. Its anti-inflammatory properties are noteworthy, as inflammation is a key factor in DBCM progression [54,55,56]. However, the effect of insulin on cardiac function is inconsistent, with echocardiographic studies often showing neutral effects on left ventricular function and CMR studies revealing minimal impact on myocardial fibrosis or steatosis [49,57]. Notably, high-dose insulin therapy, particularly in poorly managed HF, may increase the risk of adverse cardiovascular outcomes [58]. In contrast, SGLT-2i and GLP-1 receptor agonists (GLP-1RA) have shown significant reductions in major cardiovascular events, mortality, and HF hospitalization compared to insulin, with better safety profiles [59].

### 4.3. Sodium–Glucose Cotransporter-2 Inhibitors (SGLT-2i)

SGLT-2i commonly known as gliflozins, have emerged as a transformative class of oral medications for managing T2DM. Their primary role in lowering blood glucose levels is well-established; however, their cardiovascular and renal benefits, particularly concerning HF and DBCM, which extend far beyond simple glycemic control, have attracted significant attention in recent years [6,60]. These agents work by preventing the reabsorption of glucose in the kidneys, leading to increased excretion of glucose in the urine. That mechanism is additionally beneficial in reducing intravascular volume, subsequently lowering blood pressure and decreasing cardiac workload—an essential factor for patients with DBCM [8]. Also, experimental studies have shown that SGLT2 inhibitors’ protective effects seem to be related to their ability to decrease oxidative stress, intracellular calcium overload, and inflammation and to mitigate ferroptosis independent of glucose levels. This reduction in intracellular calcium and ROS appears to help preserve mitochondrial function and improve systolic function, mitigating the pathological remodeling typical of diabetic HF [61]. In addition, they modulate sodium-hydrogen exchange proteins NH1 and NH3, improving intracellular pH balance and cellular energetics [62]. NHE1 and NHE3 are Na+/H+ exchangers regulated by various kinases and signaling pathways. The NHE1 is influenced by receptors, protein kinases, and phosphatases that affect cardiac function and its hypertrophy, while NHE3 activity is modulated by phosphorylation and interactions with regulatory proteins, impacting cellular Na+ and Ca2+ balance critical for heart stability [63,64]. Furthermore, SGLT-2i exhibits anti-inflammatory properties. In the study by Wang et al. (2024), dapagliflozin significantly reduced systemic inflammation in patients with T2DM, highlighting another potential mechanism to mitigate cardiovascular risk [65]. Also, a meta-analysis in rodent models by Theofilis et al. (2022) demonstrated that SGLT2 inhibition results in a reduction of inflammatory markers, including interleukin-6 (IL-6), C reactive protein (CRP), tumor necrosis factor-α (TNF-α), and monocyte chemoattractant protein-1 (MCP-1), suggesting an anti-inflammatory effect [66].

Two major clinical trials, EMPA-REG OUTCOME and CANVAS, have confirmed the cardioprotective effects of SGLT-2 inhibitors. The EMPA-REG OUTCOME trial revealed a lower risk of MACE by 14%, a 38% decrease in cardiovascular death, and a 35% lower risk of HF hospitalization in patients with T2DM treated with empagliflozin. Similarly, the CANVAS trial showed similar results after canagliflozin administration with reduced risk of MACE and HF hospitalization by 14% and 33%, respectively [67,68]. While SGLT-2i have demonstrated efficacy in reducing hospitalization and mortality in HFpEF patients, their mechanisms remain incompletely understood, and trials have predominantly included patients with T2DM, limiting extrapolation to the DBCM population.

The effect of SGLT-2i on diastolic dysfunction in patients with HFpEF is particularly noteworthy. In most of those patients, the underlying substrate is DBCM. Imaging studies using echocardiography and/or CMR consistently have demonstrated that SGLT-2i enhances both systolic and diastolic function while reducing myocardial fibrosis [8]. In the EMPA-REG OUTCOME trial, empagliflozin led to significant improvements in LV function, including better systolic performance and reduced diastolic dysfunction [68]. Santos-Gallego et al. (2021) found that patients treated with empagliflozin experienced a 30% decrease in LV stiffness and a 15% improvement in diastolic function [69]. The DAPA-LVH study also demonstrated significantly reduced LV mass (LVM), leading to improved heart structure and diastolic function in individuals with T2DM and left ventricular hypertrophy (LVH) treated with dapagliflozin [70]. Those effects were accompanied by reduced hsCRP and insulin resistance. LVH reversal indicates overall heart-protective effects.

Empagliflozin has been shown to reduce oxidative stress in heart muscle cells exposed to high glucose levels, which helps preserve the cells’ viability and function [71,72]. Also, it ameliorates myocardial fibrosis and improves left ventricular structure and function in diabetic mice by inhibiting collagen formation via the TGF-β/Smad pathway and reducing oxidative stress through Nrf2/ARE signaling [73]. Additionally, SGLT-2i helps the heart to make metabolic adaptations by promoting the use of glucose as an energy source. This metabolic flexibility is important for maintaining the energy balance and counteracting the negative effects of glucose end-products accumulation in heart muscle cells [74]. Similarly, empagliflozin enhances cardiac metabolism by activating key pathways such as AMPK and PGC-1α, which are crucial for mitochondrial function and energy efficiency [74]. It also normalizes intracellular calcium handling in ventricular cardiomyocytes, thereby reducing arrhythmias and improving cardiac function [75].

SGLT-2i represents a significant advancement in the management of T2DM, with critical implications for the treatment of the whole range of HF. Through their diverse mechanisms of action, including glycemic control, anti-inflammatory effects, and improvements in cardiac metabolism and structure, these agents provide essential cardiovascular protection. As ongoing research continues to reveal their full potential, SGLT-2i are likely to play a greater role in DBCM therapy. We also expect more studies on their preventive effects in patients at high risk for DBCM.

### 4.4. Dipeptidyl Peptidase-4 Inhibitors

Dipeptidyl peptidase-4 (DPP-4) inhibitors, such as saxagliptin, alogliptin, sitagliptin, and linagliptin, work by enhancing incretin levels to increase insulin secretion and decrease glucagon release. While these medications are effective in managing blood glucose levels, their impact on cardiovascular outcomes, including the prevention and treatment of DBCM and HF, has been generally neutral [76,77]. Major clinical trials have not shown a significant reduction in HF incidence or cardiovascular death rate after DPP-4 inhibitors administration. Moreover, the SAVOR-TIMI 53 trial and other echocardiography-based studies revealed no substantial improvements in LV function or myocardial structure with DPP-4 inhibitor therapy [78]. Additionally, limited data from CMR imaging analysis suggest that there is no influence of those agents on myocardial fibrosis [79]. Nevertheless, DPP-4 inhibitors are considered a safe option for patients who may have contraindications to other therapies, even though they are not typically preferred for cardiovascular risk reduction [80]. According to a meta-analysis conducted by Patoulias and colleagues, DPP-4 inhibitors generally have a neutral effect on major adverse cardiovascular events, with no significant impact on the risk of heart attacks, strokes, or cardiovascular death compared to placebo in the diabetic population [81]. Therefore, DPP-4 is a safe choice for managing blood glucose in T2DM, but not necessarily the first-line therapy for DBCM.

### 4.5. GLP-1 Receptor Agonists (GLP-1 RAs)

GLP-1 RAs play a significant role in diabetes management, particularly concerning cardiovascular health. These agents not only improve glycemic control by enhancing insulin secretion and suppressing glucagon release, but they also show promise in preventing DBCM and HF [82]. Clinical trials, such as the LEADER and REWIND studies, have demonstrated that GLP-1 RAs can reduce the risk of 3-point major adverse cardiovascular events (3P-MACE) even in patients with established cardiovascular disease. The LEADER study showed that liraglutide reduced the risk of 3P-MACE by 13% and decreased cardiovascular death by 22%, while the REWIND study found that dulaglutide lowered the risk of 3P-MACE by 12%, primarily due to fewer nonfatal strokes, highlighting the cardiovascular benefits of GLP-1 receptor agonists in patients with T2DM [83]. Notably, they may prevent HF development, especially in individuals without a prior cardiovascular disease [84,85]. Additionally, GLP-1 RAs have shown beneficial effects on cardiac function. Studies using echocardiography and CMR imaging have indicated improvements in LV diastolic function, with liraglutide notably decreasing left ventricular filling pressures [86,87,88,89]. This suggests a potential role in mitigating diastolic dysfunction, a common issue in patients with DBCM. In terms of clinical outcomes, GLP-1 RAs are associated with significant reductions in cardiovascular mortality and hospitalization rates for HF [90]. On the contrary, a few trials have raised concerns about increased HF hospitalization in specific populations. However, the overall evidence is in favor of fewer cardiovascular complications.

### 4.6. Dual GIP/GLP-1 Receptor Agonists

Dual GIP and GLP-1 receptor agonists (GLP-1RA) are FDA-approved anti-diabetic drugs that combine the effects of GLP-1 receptor agonism, known to reduce cardiovascular events, with the potential benefits of GIP agonism [91]. GLP-1, an incretin hormone released by enteroendocrine L-cells in the gut, lowers blood glucose by stimulating insulin production, inhibiting α-cell function, and promoting β-cell proliferation and differentiation [91]. A meta-analysis of 23 trials confirmed that GLP-1RA and SGLT-2 inhibitors significantly reduce the risk of MACE by 13% and 11%, respectively, compared to placebo, and by 12% and 11% when compared to DPP-4 inhibitors [92]. GLP-1RAs also lower the risk of total death, hospitalization for HF, and composite renal outcomes. Paolo Basile and colleagues documented that dual GIP/GLP-1 RA may improve cardiac function, as evidenced by echocardiographic amelioration of LV function and hypertrophy [93]. Preliminary results from CMR studies indicate potential reductions in myocardial fibrosis and improved myocardial glucose uptake, which could contribute to the prevention of DBCM [94,95]. Though the long-term cardiovascular outcome data are still coming, initial studies suggest these dual agonists may offer substantial reductions in cardiovascular events [96].

### 4.7. Glitazones

The experience with glitazones highlights a cautionary tale in targeting the peroxisome proliferator-activated receptor (PPAR)-γ pathway for therapeutic purposes. While glitazones, as PPARγ agonists, improve insulin sensitivity and glucose metabolism, their significant side effects, including weight gain, fluid retention, and an increased risk of acute heart failure, have limited their clinical utility [97]. In the context of DBCM, chronic PPARγ activation may contribute to lipid accumulation and cardiomyocyte dysfunction, underscoring the need for a delicate balance between pharmaceutical modulation of PPAR pathways and cardiovascular diseases [98].

### 4.8. Physical Exercise as Adjunct Therapy

Physical exercise plays a crucial role in preventing the development of DBCM and HF in diabetic patients. It is a highly effective non-pharmacological intervention with numerous benefits, particularly in managing glucose and insulin metabolism [99]. In clinical practice, fewer patients are encouraged to exercise, and most physicians do not spend enough time to provide structured instructions, even for daily physical activity. The latter helps to prevent DBCM by inhibiting key pathological processes such as myocardial apoptosis, fibrosis, and microvascular dysfunction [100]. Moderate exercise intensity improves myocardial metabolism by upregulating glucose transporter-4 (GLUT-4) expression, enhancing glucose uptake and utilization, and activating pyruvate dehydrogenase complexes. This leads to better myocardial energy metabolism and reduces the risk of adverse cardiac remodeling, a key factor in the progression of DBCM [101]. Additionally, exercise enhances calcium regulation, protecting myocardial contractile function and preventing calcium dyshomeostasis, a major contributor to DBCM [101]. The above mechanisms can be reflected in the improvement of cardiac function through echocardiographic enhancements in ejection fraction and myocardial strain [102]. Mitochondrial function is another area where physical activity has a significant impact. It boosts mitochondrial biosynthesis and protects against diabetes-induced ultrastructural changes, promoting better energy production and reducing oxidative stress and cardiac damage [103]. Clinically, regular physical exercise is associated with a lower risk of cardiovascular events such as HF and myocardial infarction and overall mortality among T2DM patients [104,105]. Studies indicate that these benefits improve quality of life and long-term outcomes [106]. The American Diabetes Association recommends at least 150 min of moderate-intensity aerobic exercise training per week, combined with or not resistance training, to achieve optimal cardiovascular and glycemic control in diabetic patients [107].

## 5. Classical Anti-Heart Failure Therapy in Diabetic Patients

The current therapy guidelines for DBCM follow the principles of HFrEF and HFpEF therapy, tailoring to improve symptoms and prognosis. The classical pharmaceutical agents: diuretics, Angiotensin converting enzyme (ACE) inhibitors, Angiotensin II Receptor Blocker (ARBs), Angiotensin Receptor/Neprylisin Inhibitor (ARNIs), β-blockers Mineralocorticoid receptor antagonist (MRAs) and SGLT2i have been recommended in DBCM with reduced left ventricular ejection fraction (LVEF). However, some significant precautions should be taken regarding their effects on glucose regulation and the frequently co-existing diabetic nephropathy. In DBCM with preserved LVEF, SGLT2i is, up to now, the first-line therapy as an anti-diabetic agent with prognostic added value. This framework highlights the challenges faced by cardiologists to slow disease progression and prevent the side-effects of drugs in DBCM.

### 5.1. Angiotensin—Converting Enzyme Inhibitor (ACEI)—Angiotensin II Receptor Blocker (ARB)

ACEi and ARBs are widely recognized as first-line treatments for DBCM because they target the overactivation and dysregulation of the RAAS, a key mechanism connecting diabetes mellitus to cardiovascular complications [108,109]. In addition to this, they suppress cardiac inflammatory burden and fibrosis development, decelerate myocardial remodeling, and reduce LVH, thus yielding a significant improvement in systolic and, to a lesser extent, diastolic function [109]. In diabetic rats, captopril reduced end-diastolic pressure and inhibited myocardial fibrosis [110], while candesartan enhanced calcium signaling of the sarcoplasmic reticulum in cardiomyocytes [111]. Since a long time ago, clinical studies have demonstrated reduced mortality and HF hospitalization in patients with concomitant DM [112]. Recent evidence indicates that ARBs offer advantages over ACEIs due to a lower incidence of side effects such as cough and angioedema, while both classes exhibit comparable efficacy in cardiovascular outcomes [113].

In the HOPE trial, diabetic patients at high risk for cardiovascular events but with ejection fraction >40% and no signs of HF showed a 25% reduction in myocardial infarction, stroke, and cardiovascular death after ramipril therapy (RRR = 25%, 95% CI 12–36), along with a 22% decrease in HFrEF incidence [114]. In the CHARM trial, candesartan significantly reduced cardiovascular death and HF hospitalizations in patients with HFrEF (28% of participants having diabetes) (HR = 0.82; 95% CI 0.74 to 0.90) [115]. Additionally, clinical trials in diabetic kidney disease have established the efficacy of RAAS inhibitors in reducing the risks of sustained eGFR decline, kidney failure, and HF hospitalization [116]. Beyond blood pressure lowering and reducing albuminuria [117], RAAS blockade has beneficial metabolic effects, as it improves insulin resistance and glucose homeostasis [118], reduces metabolic syndrome progression, decreases inflammation and oxidative stress, and enhances FFA storage capacity in adipose tissue [119]. It is important to acknowledge that, especially in HFrEF patients, RAAS inhibitors may initially decrease GFR due to glomerular efferent arteriolar dilation, but renal function typically stabilizes with continued therapy and albuminuria drops when renal perfusion remains intact [120]. Therefore, ACEis/ARBs should not be discontinued for a modest (~30%) rise in serum creatinine [121]. In daily clinical practice, the prescription of ACEis/ARBs accompanied with close monitoring is recommended in the diabetic population, especially when cardio-renal complications are established.

### 5.2. Angiotensin Receptor/Neprylisin Inhibitor (ARNI)

ARNI, a combination of valsartan and sacubitril, provides dual benefits by inhibiting RAAS and increasing natriuretic peptide levels. In animal models, ARNI protects against DBCM by limiting inflammatory, profibrotic, and apoptotic cascades while improving LV function [122]. It also ameliorates metabolic control by enhancing insulin sensitivity via natriuretic signaling [123]. In a post-hoc analysis of the PARADIGM-HF study, diabetic patients with HFrEF treated with ARNI showed greater HbA1c reductions and delayed need for anti-diabetic therapy compared to enalapril [124]. Notably, a recent meta-analysis of 31 randomized controlled trials (RCTs) comparing ARNI with placebo showed a lower risk of new-onset diabetes in HFrEF (RR = 0.24, 95% CI: 0.12–0.50) and HFpEF (RR = 0.54, 95% CI 0.34–0.85) at the expense of risk of hypoglycemia [125]. A concomitant better glucose regulation with ARNI administration could beneficially slow down the DBCM progression. A recent study comparing the combination therapy of ARNI plus SGLT2i versus any monotherapy in diabetic patients with HFrEF favored the dual therapy attributed to the improved systolic and diastolic function and the reduced risk of HF hospitalization and cardiovascular death [126]. This underscores the importance of multi-factorial therapy for patients with DBCM. Furthermore, ARNI offers renoprotection in diabetic nephropathy by promoting natriuresis, lowering blood pressure, and mitigating inflammation and oxidative stress. It also slows the progression of glomerulosclerosis, tubulointerstitial injury, fibrosis, and podocyte damage. However, its effects on proteinuria remain uncertain, warranting further investigation [127].

### 5.3. Mineralocorticoid Receptor Antagonist (MRA)

MRAs share similar antifibrotic and anti-inflammatory effects with ACEIs, leading to improved mitochondrial function [128] and delayed cardiomyocyte apoptosis and steatosis [129]. Their beneficial effects are well-established in HFrEF. A recent individual-patient-data meta-analysis of HFpEF patients found that spironolactone reduced left atrial volume, LVM, and wall thickness while improving systolic and diastolic function [130]. However, DM was present in 32% of patients, and a high prevalence of CAD (53%) was recorded. Another meta-analysis of studies, which enrolls diabetic patients with HF, demonstrated reduced mortality after MRA therapy, compared with controls (RR = 0.78; 95% CI: 0.69–0.88; I(2) = 0%; *p* < 0.001), but there was a higher risk of hyperkalemia [131]. Although recommended by the current international recommendations for their efficacy in patients with HFpEF, and by extension, in DBCM, it is based on weak evidence [132]. Therefore, the usage of MRAs in the majority of patients with DBCM and preserved LVEF remains at the discretion of physicians who balance their pros and cons.

Finerenone, a third-generation nonsteroidal MRA, has demonstrated cardiovascular benefits in T2DM by improving fatty acid metabolism and their uptake by cardiomyocytes [133]. The FIDELITY analysis also revealed a reduced HF-related hospitalization rate in patients with T2DM and CKD [134]. Finerenone reduces cardiomyocyte apoptosis, restores autophagy, mitigates oxidative stress, and prevents mitochondrial dysfunction in DBCM by modulating signaling pathways such as PPARα/CD36 and PI3K/Akt/eNOS while also potentially influencing microRNA regulation and intestinal microbiota [135]. More studies focusing on patients with DBCM will clarify their efficacy and safety.

### 5.4. B-Blockers

In DBCM, excessive sympathetic activation [136] makes β-blockers effective by protecting against catecholamine toxicity, reducing myocardial oxygen consumption, and improving cardiac metabolism [137]. Based on old large-scale clinical trials, b-blockers—carvedilol, metoprolol succinate, and bisoprolol—have been shown to reduce morbidity and mortality in patients with diabetes and HFrEF, achieving slightly less degree of benefit compared to non-diabetic patients [138]. The American Heart Association recommends their use in diabetic patients with HF, but they do not distinguish the DBCM population [90]. In the case of HFpEF, there are inconsistent results regarding the efficacy of β-blockers on clinical outcomes, ambiguating their general use unless they are prescribed for specific co-morbidities (e.g., atrial fibrillation) [139]. Notably, the PRESERVE-HF trial demonstrated that b-blockers discontinuation may enhance functional capacity in patients with HFpEF and chronotropic incompetence [140]. In contrast, a recent prespecified analysis of the DELIVERY trial in patients with HFpEF treated with dapagliflozin found that 83% of participants were on b-blockers, and their use was not associated with a higher risk of worsening HF or cardiovascular death [141]. Regarding the majority of DBCM patients with preserved ejection fraction, more dedicated studies are required to evaluate the indications for β-blockers in this population. Moreover, β-blockers may be underutilized in T2DM due to concerns about masking hypoglycemia symptoms [142], as well as their potential to inhibit compensatory hepatic glucose production [143]. An adaptation of the anti-diabetic regimen may be considered. Moreover, β-blockers-receivers with pre-diabetes are at an increasing risk for DM onset [137], along with decreased high-density lipoprotein (HDL) levels and increased triglycerides levels [144]. Based on experimental and clinical data, carvedilol, a combined β1/β2/α1 antagonist, could be actually beneficial for DBCM, enhancing glycemic control, reducing oxidative stress, and improving LVEF [142]. Overall, the need for β-blockers usage in patients with DBCM with reduced LVEF is unambiguous, while in the case of preserved LVEF, their administration may be considered only when other co-morbidities make them necessary with well-controlled precautions.

### 5.5. Diuretics

Their impact on mortality remains unclear, though they are essential for symptomatic relief and decongestion in patients with acute HF [145]. Loop diuretics, such as furosemide, are generally preferred. For those with refractory edema unresponsive to loop diuretics alone, metolazone can be added. A significant issue that is not uncommon in DBCM is the co-existence of diabetic nephropathy. The latter may influence their response to diuretics, and even a small reduction in plasma volume may worsen kidney function. That adverse effect may be overcome by the additional administration of SGLT2i, which reduces the required doses of diuretics [146]. More research is necessary to pinpoint the least effective dose of diuretics to relive dyspnea in DBCM with preserved LVEF.

### 5.6. Myosin Inhibitors

Mavacamten, a selective allosteric inhibitor of cardiac myosin, acts by reducing the excessive actin-myosin cross-bridge formation, thereby alleviating hypercontractility and restoring normal diastolic function [147]. This mechanism makes it potentially relevant in conditions like DBCM, where impaired relaxation and diastolic dysfunction play a key role [148]. By targeting the underlying molecular abnormalities, mavacamten has the potential to improve cardiac compliance and mitigate the progression of HF in diabetic patients. However, currently, no experimental or clinical studies are investigating their potential effects on diabetic cardiomyopathy.

## 6. Novel Therapies

Most data regarding the effects of novel therapies on DBCM development and progression are derived from pre-clinical studies and, therefore, require clinical validation.

### 6.1. Nucleotide-Binding and Oligomerization Domain-like Receptor Family Pyrin Domain-Containing 3 (NLRP3) Inflammasome

Nucleotide-binding oligomerization domain-like receptor proteins, particularly the NLRP3 inflammasome, play a crucial role in DBCM and other diabetic complications [149]. The NLRP3 inflammasome is a protein complex (approximately 700,000 Da) that detects stress signals via pattern recognition receptors and triggers the release of inflammatory cytokines like IL-1β, IL-18, and IL-33, leading to pyroptosis, a form of cell death [150]. Increased expression of NLRP3 inflammasome has been observed in cardiac tissue of DBCM patients [151]. Experimental studies show that Taohuajing, derived from Persicae semen, reduces ROS and malondialdehyde, boosts antioxidant defenses like glutathione peroxidase and superoxide dismutase (SOD), and inhibits NLRP3 inflammasome activation [151], as a potential treatment for DBCM (Table 2). NLRP3 gene silencing in rat models has reversed myocardial damage, decreased fibrosis and apoptosis, and improved left ventricular function [149]. Additionally, canagliflozin reduces NLRP3 activation and ROS production in diabetic endothelial cells [150]. Currently, there are no clinical studies examining the effect of NLRP3 inflammasome inhibition in DBCM.

### 6.2. Natural Products (Herbal Medicine)

Traditional herbal derivatives have been repeatedly tested in T2DM treatment, as food complements, or for new drug development. In preclinical studies, many natural products have exerted anti-diabetic, antioxidant, and anti-inflammatory actions, usually through the regulation of NF-κB and Nrf2 pathways (Table 3) [151]. Recent experimental research emphasizes the role of endoplasmic reticulum stress (ERS) in DBCM progression, with several Traditional Chinese medicine (TCM) ingredients and formulas, such as Astragalus polysaccharide, alleviating ERS-related cardiac damage [152]. Formulas like Tongluo Nourishing Yin, Si-Miao-Yong-An decoction, Tanshinone IIA, Triptolide, Cola nitida seeds, Matrine, and qi-nourishing yin-activating blood circulation formulas have been shown to improve LV function, reduce hypertrophy, fibrosis, and inflammation, and enhance diastolic function only in experimental models [153,154,155,156,157]. Natural products like Guan Xin Dan Shen and Cyclovirobuxine D enhance cardiac function by activating antioxidant pathways (Akt/Nrf2), while Lycium Chinese leaf extract and Cyclocarya paliurus ethanol extract reduce blood glucose levels and alleviate pathological cardiac damage [158]. TCM ingredients like ginseng fruit saponins, ginsenosides, Shengmai San, and gypenosides prevent damage to diabetic myocardium via several mechanisms with therapeutic potential for DBCM [159,160].

Curcumin, extracted from turmeric (Curcuma longa), protects cardiomyocytes by alleviating lipotoxicity through ERS modulation [160]. It alleviates cardiac remodeling in diabetic animal models by downregulating PKC-α and PKC-β2 and inhibiting the NF-κB and MAPK pathways [10]. Curcumin activates the protein kinase B (AKT)/Nrf2/antioxidant response element signaling pathway, promoting the transfer of Nrf2 into the nucleus, leading to increased expression of antioxidant factors like glutamate-cysteine ligase catalytic subunit, reducing the accumulation of ROS and mitigated mitochondrial damage [161,162]. Moreover, a recent study found that ginger and omega-3 fatty acids significantly improved cardiac function, reduced oxidative damage, and suppressed inflammation and apoptosis in diabetic rats [163]. While all those findings are promising, they are derived from experimental studies, and no clinical trials have been conducted to date. Furthermore, due to the complex nature of these formulations, there are significant challenges in studying their pharmacodynamics and pharmacokinetics, which complicates the evaluation of the precise concentration of the pure substance in the pharmaceutical context.

### 6.3. Aldose Reductase Inhibitors

Activation of the aldose reductase pathway by hyperglycemia leads to sorbitol accumulation, oxidative stress, deposition of AGEs and PKC, inflammation, and fibrosis [164]. Aldose reductase inhibitors (ARIs) reduce oxidative stress and may prevent DBCM progression (Table 4) [164]. The ongoing ARISE-HF trial is testing AT-001, a novel ARI, to improve cardiac function in patients with T2DM by addressing the metabolic abnormalities contributing to DBCM [165]. Preclinical studies showed that AT-001 improved diastolic function and reduced fibrosis and hypertrophy in diabetic mice [166].

### 6.4. Antioxidants

NADPH oxidase plays a key role in promoting cardiomyocyte hypertrophy and fibrosis and activating profibrotic pathways (Table 4) [167,168]. Preclinical studies have shown that antioxidants like SOD, sirtuin 3, AMPK activators, and Nrf2 activators such as sulforaphane, curcumin, and resveratrol reduce oxidative damage, improve mitochondrial function, and prevent cardiac fibrosis and hypertrophy [169]. Mitochondrial antioxidants, including Coenzyme-Q10, mito-TEMPO, Peroxiredoxin-3 (Prx-3), and N-acetyl-L-cysteine, protect against oxidative stress in DBCM by enhancing antioxidant defenses [21]. Coenzyme-Q10 has been shown to reduce LV mass and collagen deposition, while mito-TEMPO and Prx-3 protect cardiomyocytes from hyperglycemia-induced damage [170,171]. Additionally, compounds like epigallocatechin-3-gallate (EGCG), costunolide, and dexmedetomidine reduce oxidative stress and inflammation, offering potential therapeutic benefits for DBCM [172].

### 6.5. Mitochondrial Disorder Treatment

Experimental studies suggest that ubiquitin-specific protease 28 (USP28) and USP7 are potential therapeutic targets for DBCM (Table 4) [173,174]. Further studies showed that FGF1ΔHBS treatment improves cardiac function by activating AMPK and reducing mitochondrial dysfunction in diabetic mice [175]. Downregulation of caveolin 3 (CAV3) in DBCM leads to mitochondrial dysfunction and cardiomyocyte apoptosis, while CAV3 overexpression restores mitochondrial function and reduces oxidative stress [176].

### 6.6. Autophagy Dysregulation

Autophagy dysregulation leads to cardiomyocyte apoptosis and fibrosis in DBCM (Table 4) [177]. Mitochondrial-targeted therapies like ALDH2 activators also support autophagy by reducing oxidative stress [178]. Preclinical studies with agents like metformin, liraglutide, resveratrol, and autophagy-related gene 7 (ATG7) overexpression demonstrated promising reduction of oxidative stress and improvement in cardiac outcomes in DBCM [179,180,181].

### 6.7. Flavonoids

Flavonoids, a diverse group of plant-based polyphenolic compounds, exert cardioprotective effects in DBCM by reducing oxidative stress, mitigating chronic inflammation and ROS production, enhancing insulin sensitivity, and regulating glucose and lipid metabolism (Table 3) [182,183]. Flavonoids like myricitrin, taxifolin, and luteolin have been shown to activate Nrf2 while inhibiting NF-κB, resulting in reduced fibrosis, hypertrophy, and apoptosis in diabetic hearts [184,185]. Myricitrin activates the protein kinase B pathway and modulates extracellular signal-regulated kinase signaling, improving mitochondrial function and reducing oxidative stress [186]. Taxifolin, also known as dihydroquercetin, enhances antioxidant enzyme activity, reduces myocardial apoptosis, and regulates the Janus kinase/signal transducer and activator of the transcription 3 pathway, improving diastolic function in diabetic mice [185]. Icariin, a flavonoid from Epimedium, alleviates insulin resistance, improves LV function, and reduces cardiac fibrosis by inhibiting the transforming growth factor-beta 1/Smad signaling pathway in diabetic rats [187]. Galangin and wogonin provide antioxidant and anti-inflammatory effects, reducing apoptosis and oxidative damage in diabetic hearts by modulating pathways like NF-κB and inducible NO synthase [188]. Flavonoids, such as naringin and naringenin, regulate key oxidative stress pathways like Nrf2, NF-κB, and NADPH oxidase, thus showing promise in reducing myocardial apoptosis and improving cardiac function [189]. Most of these findings are derived from experimental studies, with quercetin and curcumin being the most extensively researched flavonoids in the context of DBCM.

### 6.8. MicroRNAs

MicroRNAs (miRNAs), small noncoding RNAs, regulate gene expression and play crucial roles in DBCM (Table 4) [190,191]. Aberrant miRNA expression, such as miR-133a and MMP9, is linked to oxidative stress, inflammation, fibrosis, apoptosis, and mitochondrial dysfunction in DBCM, making them promising targets for diagnosis and treatment [191]. Specific miRNAs, including miR-30d, miR-21-3p, miR-145, and miR-9, influence pathways like autophagy, calcium metabolism, and pyroptosis [192,193]. KCNQ1OT1 promotes inflammation by regulating the miR-181a-5p/PDCD4 pathway, suggesting a potential therapeutic target [194]. Preclinical studies indicate promising therapeutic potential of histone deacetylase inhibitors and miRNA-targeting therapies in reducing oxidative stress, inflammation, and fibrosis in DBCM, though human trials are still needed [195,196].

## 7. Materials and Methods

We conducted a literature search in the English language for publications in the MED-LINE and EMBASE, Web of Science, Cochrane, and Google Scholar databases from 1990 to October 2024. The following search terms for titles and abstracts, including Medical Subject Headings (MeSH), were used: diabetic cardiomyopathy, heart failure, SGLT2i, pharmaceutical therapy, novel therapies, and anti-diabetic therapy. Five investigators (EK, SS, MK, EL, and AM) independently performed the literature search. We included experimental studies, in vitro and in vivo, and clinical studies as well. We further limited our literature search by setting the following exclusion criteria: studies with full text unavailable, published in languages other than English, and conference abstracts. The reference lists of the identified articles were checked for any additional relevant articles, especially among reviews.

## 8. Discussion

The pharmaceutical therapy of DBCM represents a significant challenge due to its complex, multifactorial pathophysiology requiring nuanced pharmaceutical intervention to address clinical manifestations. Central mechanisms, including hyperglycemia-induced oxidative stress, inflammation, lipotoxicity, microvascular dysfunction, and neurohormonal abnormalities, contribute to progressive cardiac dysfunction, initially diastolic and eventually systolic. These processes highlight the inadequacies of traditional HF therapies, which are often applied indiscriminately to diabetic and non-diabetic populations. Moreover, there is robust evidence of the prognostic value of HF therapy in the case of HFrEF rather than HFpEF. The unique pathophysiological drivers of DBCM and the LVEF could regulate the essential therapy for improving patient outcomes.

An essential element of DBCM management is the optimization of glycemic control along with the minimization of cardiovascular risk. SGLT-2i has emerged as a transformative first-line therapy. The existing robust evidence supports their cardiovascular and renal benefits in addition to their glucose-lowering effects. Large clinical trials such as EMPA-REG OUTCOME and CANVAS have demonstrated significant reductions in HF hospitalizations and cardiovascular mortality, indicating their role in DBCM management. Furthermore, metformin, an old, anti-diabetic medication with a beneficial cardiovascular profile, remains an essential part of the anti-diabetic regimen. Its combination with SGLT-2i could exert additional effects on the amelioration of myocardial energetics and diastolic dysfunction, especially in HfpEF, where therapeutic options have been limited. It is also important to underscore the emerging role of GLP-1 agonists and dual GIP/GLP-1 receptor agonists in DBCM management. These agents not only enhance glycemic control but also exert anti-inflammatory, antifibrotic, and cardioprotective effects. Clinical trials such as LEADER and REWIND enrolling diabetic patients have demonstrated reduced MACE, suggesting their potential utility in mitigating the progression of DBCM. Physical exercise, as an adjunct therapy, has shown benefits in reversing cardiac remodeling, improving diastolic function, and reducing HF risk. It is always a complementary therapy to pharmaceutical interventions.

ACEIs/ARBs and ARNIs also hold promise in DBCM treatment. By targeting neurohormonal dysregulation, these agents mitigate fibrosis, inflammation, and myocardial remodeling. Post-hoc analyses of the PARADIGM-HF trial indicate improved glucose regulation and reduced HF hospitalizations in diabetic populations, further advocating for their use in DBCM patients with HFrEF. Except for SGLT-2i, the prognostic benefits from traditional HF therapies in DBCM with preserved LVEF are less supported, while several cautions should be taken in routine clinical practice. Beta-blockers may exacerbate glucose dysregulation and mask hypoglycemic symptoms, limiting their utility in DBCM. Similarly, MRAs and diuretics provide symptomatic relief but lack robust evidence for improving long-term outcomes in DBCM, particularly in HFpEF.

Emerging therapies, including natural products, NLRP3 inflammasome inhibition, microRNA modulators, aldose reductase inhibitors, and mitochondrial-targeted antioxidants, represent promising avenues for addressing the underlying molecular derangements in DBCM. Furthermore, their clinical translation faces challenges such as bioavailability and off-target effects, which necessitate further investigation before widespread adoption.

## 9. Conclusions

Although conventional HF therapy is widely used in diabetic populations, its efficacy in DBCM remains insufficiently evidenced. A significant part of the underlying pathophysiology is out of the spectrum of the current pharmaceutical therapy, and clinical trials dedicated to DBCM are deemed necessary. Established therapies for HFrEF are highly recommended in DBCM with reduced LVEF. SGLT2i and, to a lesser extent, ARNI may improve prognostication in DBCM with preserved LVEF. Classical anti-diabetic therapies, including metformin, SGLT-2i inhibitors, DPP-4 inhibitors, and the dual GIP/GLP-1 RA, may play significant roles in improving cardiac function and clinical outcomes in patients with established DBCM. The integration of physical exercise into diabetes management further enhances cardiovascular health and improves clinical outcomes. Novel therapies have been, up to now, only assessed in preclinical studies, and much more should be performed to establish their clinical efficacy. Tailoring therapy to individual pathophysiology is essential for optimizing both glycemic control and long-term heart health in diabetic patients.

## Figures and Tables

**Table 1:** Overview of classical anti-diabetic therapies in the mechanisms of action, prevention of diabetic cardiomyopathy, cardiac function improvement, and clinical outcomes.

Medication	Mechanism of Action	Prevention of Diabetic Cardiomyopathy/Heart Failure	Improvement of Cardiac Function	Effects on Clinical Outcomes
Metformin	↓ hepatic glucose production, ↑ insulin sensitivity, AMPK activation	↓ incidence HF, CV events ↓ oxidative stress and inflammation	↑ LV systolic and diastolic function, ↓ LV mass, ↓ fibrosis	↓ HF and CV events, especially in established heart disease
Insulin	↑ glucose uptake in muscle and fat cells ↑ myocardial glucose uptake and anti-inflammatory cytokines ↓ risk of cardiac dysfunction and glucose toxicity	↓ risk of hyperglycemia-induced oxidative stress and inflammation ↓ incidence of DBCM and HF ↑ myocardial function and insulin sensitivity	←→ LV function, ←→ myocardial fibrosis (CMR)	↓ DBCM risk, May worsen HF and CV events
SGLT-2 Inhibitors	↓ glucose reabsorption in kidneys ↑ glucose excretion ↓ blood pressure and intravascular volume	↓ risk of HF and CV death, ↑ myocardial energy efficiency	↑ systolic and diastolic function, ↓ myocardial fibrosis	↓ MACE, mortality, and HF hospitalization
GLP-1 Agonists	↑ insulin secretion ↓ glucagon release and ↓ gastric emptying	↓ risk of HF and CV death	↑ LV diastolic function ↓ LV filling pressures	↓ MACE and all-cause mortality
DPP-4 Inhibitors	↑ incretin levels to enhance insulin secretion, ↓ glucagon release	Neutral effect on HF and cardiomyopathy prevention	No significant improvement in LV function or myocardial structure in echo or CMR studies	←→CV outcomes, ←→ HF incidence
Dual GIP/GLP-1 Receptor Agonists	↑ insulin production, inhibits α-cell function, ↑ β-cell proliferation, ↑ glucose and lipid metabolism	↓ MACE ↓ myocardial fibrosis	↑ LV function ↑ LV hypertrophy and fibrosis (preliminary studies)	↓ CV events and HF hospitalization
Physical Exercise	↑ glucose uptake in muscle and fat cells, ↑ GLUT-4 expression, ↑ mitochondrial and calcium function	↓ myocardial apoptosis, fibrosis, and microvascular dysfunction	↑ ejection fraction, myocardial strain, ↓ fibrosis, ↑ mitochondrial function	↓ risks of HF, MI, and overall mortality

AMPK—AMP-activated protein kinase; CMR—Cardiovascular magnetic resonance; CV—Cardiovascular; DBCM—Diabetic cardiomyopathy; GLUT-4—Glucose transporter type 4; HF—Heart failure; LV—Left ventricle, MACE—major adverse cardiovascular events; MI—myocardial infarction; ↑—increase; ↓ decrease; ←→—no change.

**Table 2:** Novel DBCM therapies: NLRP3 inflammasome suppressors.

Medications	Mechanisms	Potential Cardio-Protective Effects
Taohuajing	↓ ROS and MDA production	↑ GSH-Px and SOD, ↓ pro-inflammatory cytokines, ↓ NLRP3 inflammasome activation, ↓ oxidative stress and inflammation
NLRP3-miRNA	NLRP3 gene silencing	↓ inflammasome activation, ↓ interstitial fibrosis, and cardiac cell apoptosis, improves LV systolic and diastolic function, reverses myocardial damage
Rapamycin	Autophagy activation	↓ NLRP3 inflammasome formation, ↓ inflammation, ↓ myocardial ischemia-reperfusion-induced inflammation and damage
Canagliflozin	↓ NLRP3 inflammasome activation	↓ ROS production in diabetic endothelial cells, ↓ oxidative stress and inflammation in diabetic endothelial cells
ALDH2	↓ harmful metabolites (4-HNE)	↓ mitochondrial oxygen free radicals, ↓ NLRP3 inflammasome activation, protects against myocardial ischemia/reperfusion injury

4-HNE: 4-Hydroxy-2-nonenal; ALDH2: Aldehyde Dehydrogenase 2; GSH-Px: Glutathione Peroxidase; LV: Left Ventricular; MDA: Malondialdehyde; NLRP3: Nucleotide-binding and oligomerization domain-like receptor family pyrin domain-containing 3; ROS: Reactive Oxygen Species; SOD: Superoxide Dismutase; ↑: increase; ↓: decrease.

**Table 3:** Pre-clinical data about novel DBCM therapies: Natural products and flavonoids.

Medications	Mechanisms	Potential Cardio-Protective Effects
Natural products (herbal medicine)		
Tongluo Nourishing Yin Formula/Si-Miao-Yong-An Decoction	↓ Oxidative stress, inflammation, apoptosis, and fibrosis	↑ LV function ↓ hypertrophy, fibrosis, and inflammation
Astragalus Polysaccharide	Inhibits ERS-related pathways Modulates glucose metabolism via GLUT-4, ↓ PPAR-α pathway	↓ cardiac fibrosis and apoptosis, ↑ antioxidant effects→ ↑cardiac function
Aloe-emodin Derivative	↓ NLRP3 inflammasome-mediated pyroptosis pathway	↓ inflammation
Qigui Qiangxin Mixture	Activates PI3K-AKT signaling pathway	↓ apoptosis ↑cardiac function
Rhynchophylline	Regulates calcium homeostasis Prevents mitochondrial calcium overload,	↑cardiac function ↓ myocardial damage
Guan Xin Dan Shen Formulation	Activates Akt/Nrf2 signaling pathway	↓ apoptosis, ↑ cardiac function
Cyclovirobuxine D	Activates Nrf2 signaling ↓ oxidative stress, restores mitochondrial membrane potential ↑ antioxidant enzymes	↑ cardiac function
Lycium Chinese Leaf Extract	↓ oxidative stress, inflammation, apoptosis, and fibrosis	↑ cardiac function, restores cardiac structural integrity
Panax Notoginseng Saponin	↓ lipid accumulation and oxidative stress ↑ mitochondrial function	↑ cardiac function
Cyclocarya Paliurus Ethanol Leaf Extract	Inhibits NF-κB signaling, activates PI3K/Akt pathway	↓ fibrosis, hypertrophy, ↑ lipid profiles and glucose levels
Resveratrol	Regulates ERS pathways, activates AMPK/AKT cascades, ↑ Nrf2 Modulates autophagy, ↓ oxidative stress,	↑ cardiac function, ↓ hypertrophy, apoptosis
Puerarin	↓ ERS-related proteins ↓ blood glucose, ↓ insulin resistance	Cardioprotective
Astragaloside IV	Inhibits ERS-related apoptosis in cardiomyocytes	↓ myocardial damage in DBCM
Mangiferin	↓ ERS markers (IRE1 and JNK)	↓ myocardial fibrosis, ↑ cardiac function
Ginseng Fruit Saponins/Ginsenoside Rg1	Regulates ERS-related proteins (GRP78, CHOP)	↓ apoptosis and fibrosis, ↑ cardiac function
Gypenosides	↓ ROS-mediated activation of the NLRP3 inflammasome	↓ myocardial damage in DBCM
Shengmai San	Activates SIRT1/AMPK/PGC-1α pathway Stabilizes mitochondrial structure	↓ myocardial hypertrophy and diastolic dysfunction
Curcumin	Activates AKT/Nrf2/ARE pathway ↓ ROS accumulation, ↑ mitochondrial function, activates autophagy, ↓ oxidative stress,	↓ cardiac remodeling
Tanshinone IIA	↓ ERS-mediated apoptosis and fibrosis	↓ myocardial fibrosis, ↑ cardiac function
Triptolide	↓ NF-κB/IL-1β and NF-κB/TNF-α pathways	↓ myocardial fibrosis and hypertrophy, ↑ cardiac function
Cola Nitida	Improves oxidative stress markers	↓ ACE activity, preserves myocardial structure
White Mulberry Root-Bark/Ginkgo Biloba Leaves	Targets ERS pathways (PERK/eIF2α and JNK)	↓ cardiac apoptosis, fibrosis
Ginkgo Biloba Extract	Inhibits mitochondrial permeability transition pore	↓ oxidative stress, protects against diabetic-induced cardiac damage
Matrine/Cryptotanshinone	Inhibits STAT3 pathway and MMP-9 expression	↓ cardiac fibrosis
Broccoli Sprout Extract/Garlic/Dietary Phenolic Acids	Activate Nrf2 transcription	Prevents cardiac dysfunction
Dendrobium Officinale	↓ NF-κB-mediated inflammation	Attenuates diabetic heart injury
Yunpi-Huoxue-Sanjie	↑ autophagy through FoxO1 signaling pathway	↑ cardiac function, ↓ fibrosis
Notoginsenoside R1	Activates estrogen receptor α, Akt-Nrf2 signaling, inhibits TGFβ pathway ↓ oxidative stress, apoptosis, and fibrosis	↑ cardiac function,
**Flavonoids**		
Myricitrin	↑ Akt pathway, modulates ERK signaling, ↑ mitochondrial function ↓ oxidative stress and apoptosis	
Taxifolin	↑ antioxidant enzyme activity, regulates JAK/STAT3 pathway	↓ myocardial apoptosis, ↑ diastolic function
Icariin	↓ TGF-β1/Smad pathway	↑ LV function, ↓ fibrosis
Galangin	Modulates NF-κB and iNOS pathways	↓ apoptosis and oxidative damage in diabetic hearts
Wogonin	↓ oxidative damage and inflammation via NF-κB and iNOS pathways	↓ apoptosis and oxidative damage
Naringin and naringenin	Regulate Nrf2, NF-κB, and NOX pathways to ↓ oxidative stress	↓ myocardial apoptosis, ↑ cardiac function
Hesperetin	Modulates oxidative stress pathways	↑ cardiac function prevents fibrosis
Cyanidin-3-glucoside and delphinidin	Regulate oxidative stress	↑ cardiac function, prevents fibrosis
Phloretin	Targets Keap1/Nrf2 pathway	↓ oxidative stress
Quercetin	Promotes nuclear translocation of Nrf2, inhibits pyroptosis	↓ myocardial fibrosis and apoptosis
Nobiletin, baicalein, and fortunellin	↓ JNK and p38 MAPK pathways, ↑ antioxidant defenses via PI3K/Akt	Protects against cardiac damage

ACE: Angiotensin-Converting Enzyme; AKT: Protein Kinase B; AMPK: AMP-Activated Protein Kinase; ARE: Antioxidant Response Element; DBCM: Diabetic Cardiomyopathy; ERS: Endoplasmic Reticulum Stress; ERK: Extracellular Signal-Regulated Kinase; FoxO1: Forkhead Box O1; GLUT-4: Glucose Transporter Type 4; GRP78: Glucose-Regulated Protein 78; IL-1β: Interleukin-1 Beta; iNOS: Inducible Nitric Oxide Synthase; IRE1: Inositol-Requiring Enzyme 1; JAK: Janus Kinase; JNK: c-Jun N-terminal Kinase; Keap1: Kelch-like ECH-Associated Protein 1; LV: Left Ventricular; MAPK: Mitogen-Activated Protein Kinase; MMP-9: Matrix Metalloproteinase 9; NLRP3: Nucleotide-binding and oligomerization domain-like receptor family pyrin domain-containing 3; NOX: NADPH Oxidase; Nrf2: Nuclear Factor Erythroid 2-Related Factor 2; PERK: PKR-Like Endoplasmic Reticulum Kinase; PGC-1α: Peroxisome Proliferator-Activated Receptor Gamma Coactivator 1-Alpha; PI3K: Phosphoinositide 3-Kinase; PPAR-α: Peroxisome Proliferator-Activated Receptor Alpha; ROS: Reactive Oxygen Species; SIRT1: Sirtuin 1; Smad: Family of Signal Transducers and Activators of Transcription; STAT3: Signal Transducer and Activator of Transcription 3; TGF-β1: Transforming Growth Factor Beta 1; TNF-α: Tumor Necrosis Factor Alpha; ↑: increase; ↓: decrease.

**Table 4:** Novel DBCM therapies: ARIs, antioxidants, mitochondrial disorder treatments, autophagy dysregulation, and MicroRNAs. With the exception of the ARISE-HF trial, all other data were derived from pre-clinical studies.

Medications	Mechanisms	Potential Cardio-Protective Effects
Aldose reductase inhibitors		
AT-001 (ongoing clinical ARISE-HF trial)	Inhibits aldose reductase ↓ oxidative stress	↑ cardiac function, diastolic function and cardiac efficiency ↓ heart failure progression, myocardial fatty acid oxidation, cardiac fibrosis, and hypertrophy
Benzofuroxane	↑ expression of antioxidant and protective proteins (SIRT1, MnSOD, eNOS, FOXO-1) ↑ energy consumption, ↓ oxidative stress, ↓ DNA damage, ↑ antioxidant pathways	
SIRT1 inhibition		Improves cardiac efficiency
Sorbinil	↑ autonomic nervous system function	↑ cardiac function, resting, and maximal cardiac output
**Antioxidants—Preclinical data**		
SOD	Antioxidant enzyme that scavenges ROS	↓ oxidative damage, cardiac fibrosis, and hypertrophy
Sirtuin 3	↑ mitochondrial function	↓ oxidative stress damage, mitochondrial dysfunction
Nrf2 activators	↑ antioxidant defenses	↓ oxidative damage, cardiac fibrosis, and hypertrophy
Coenzyme-Q10	↑ mitochondrial antioxidant	↓ LV mass and collagen deposition
Mito-TEMPO	↑ mitochondrial ROS scavenger	↓ hyperglycemia-induced damage of cardiomyocytes
Peroxiredoxin-3	↑ mitochondrial antioxidant	↓ hyperglycemia-induced damage of cardiomyocytes
N-acetyl-L-cysteine	↑ antioxidant defense	↓ ROS, ↓ cardiac fibrosis
Zinc	↑ insulin signaling	↓ cardiac remodeling and fibrosis
Epigallocatechin-3-gallate	↓ oxidative stress and inflammation	Modulates autophagy pathways, protects cardiac cells
Costunolide	↓ oxidative stress and inflammation	
Dexmedetomidine	↓ oxidative stress and inflammation	
**MicroRNAs—Preclinical data**		
miR-133a and MMP9	↓ oxidative stress, ↓ inflammation, and mitochondrial dysfunction	
miR-30d	Modulates autophagy, calcium metabolism, and pyroptosis	↓ fibrosis
miR-21-3p	Influences autophagy and pyroptosis pathways	↓ inflammation and fibrosis
miR-145	Modulates autophagy and inflammation	↓ inflammation and fibrosis
miR-9	Influences autophagy, calcium metabolism, and pyroptosis	↓ cardiac damage
HDACIs	↓ oxidative stress, inflammation	↓ fibrosis
**Mitochondrial disorders treatments—Preclinical data**		
USP28 and USP7	↑ PPARα stability, ↑ lipid metabolism regulation, ↑ Mfn2 for mitochondrial fusion	↑ cardiac function
Bmal1 overexpression	↑ mitochondrial function, ↓ Ca^2+^ overload	↓ cardiac injury
FGF1ΔHBS	↑ AMPK activation, ↓ mitochondrial dysfunction	↑ cardiac function
Caveolin 3 overexpression	↑ mitochondrial function, ↓ oxidative stress	↓ cardiomyocyte apoptosis
Mesenchymal stem cells, Pioglitazone, Exendin-4, iPSCs	Target ↓ oxidative stress and ↓ fibrosis	↑ cardiac function
**Autophagy Dysregulation—Preclinical data**		
Neuregulin-4	Reactivates autophagy through the ↑ AMPK/mTORC1 pathway	↓ cardiomyocyte apoptosis, ↓ fibrosis, ↑ cardiac function
ALDH2 activators	↓ oxidative stress, supports autophagy	↓oxidative damage
ATG7 overexpression	↑ autophagy, ↓ oxidative stress	↑ cardiac function
Polyphenols	Modulates autophagy pathways to ↓ oxidative stress	↑ autophagy
Berberine	↓ oxidative stress, ↑ autophagy	↑ autophagy in the heart

ALDH2: Aldehyde Dehydrogenase 2; AMPK: AMP-Activated Protein Kinase; ATG7: Autophagy-Related Protein 7; Bmal1: Brain and Muscle ARNT-Like Protein 1; Ca^2+^: Calcium Ion; Caveolin 3: Caveolin Protein 3; DNA: Deoxyribonucleic Acid; eNOS: Endothelial Nitric Oxide Synthase; FGF1ΔHBS: Fibroblast Growth Factor 1 with Deletion in Heparin-Binding Site; FOXO-1: Forkhead Box O1; HDACIs: Histone Deacetylase Inhibitors; iPSCs: Induced Pluripotent Stem Cells; Mfn2: Mitofusin 2; miR: MicroRNA; Mito-TEMPO: Mitochondria-Targeted TEMPOL; MnSOD: Manganese Superoxide Dismutase; MMP9: Matrix Metallopeptidase 9; mTORC1: Mechanistic Target of Rapamycin Complex 1; N-acetyl-L-cysteine: Antioxidant and Glutathione Precursor; Neuregulin-4: Growth Factor Protein; Nrf2: Nuclear Factor Erythroid 2-Related Factor 2; PPARα: Peroxisome Proliferator-Activated Receptor Alpha; ROS: Reactive Oxygen Species; SIRT1: Sirtuin 1; SOD: Superoxide Dismutase; USP: Ubiquitin-Specific Peptidase; ↑: increase; ↓: decrease.

## Data Availability

The literature cited in this review article was sourced from MEDLINE and EMBASE, Web of Science, Cochrane, and Google Scholar databases. All referenced publications are publicly available through these databases, ensuring accessibility and transparency in data availability.

## References

[1] Lee W.S., Kim J. (2017). Diabetic cardiomyopathy: Where we are and where we are going. Korean J. Intern. Med..

[2] Hopf A.E., Andresen C., Kötter S., Isić M., Ulrich K., Sahin S., Bongardt S., Röll W., Drove F., Scheerer N. (2018). Diabetes-Induced Cardiomyocyte Passive Stiffening Is Caused by Impaired Insulin-Dependent Titin Modification and Can Be Modulated by Neuregulin-1. Circ. Res..

[3] Lorenzo O., Picatoste B., Ares-Carrasco S., Ramírez E., Egido J., Tuñón J. (2011). Potential role of nuclear factor κB in diabetic cardiomyopathy. Mediat. Inflamm..

[4] Lambadiari V., Dimitriadis G., Kadoglou N.P.E. (2018). The impact of oral anti-diabetic medications on heart failure: Lessons learned from preclinical studies. Heart Fail. Rev..

[5] McDonagh T.A., Metra M., Adamo M., Gardner R.S., Baumbach A., Böhm M., Burri H., Butler J., Čelutkienė J., Chioncel O. (2021). 2021 ESC Guidelines for the diagnosis and treatment of acute and chronic heart failure. Eur. Heart J..

[6] Pocock S.J., Wang D., Pfeffer M.A., Yusuf S., McMurray J.J., Swedberg K.B., Ostergren J., Michelson E.L., Pieper K.S., Granger C.B. (2006). Predictors of mortality and morbidity in patients with chronic heart failure. Eur. Heart J..

[7] Cannata F., Vadala G., Russo F., Papalia R., Napoli N., Pozzilli P. (2020). Beneficial Effects of Physical Activity in Diabetic Patients. J. Funct. Morphol. Kinesiol..

[8] Huang K., Luo X., Liao B., Li G., Feng J. (2023). Insights into SGLT2 inhibitor treatment of diabetic cardiomyopathy: Focus on the mechanisms. Cardiovasc. Diabetol..

[9] Boudina S., Sena S., Sloan C., Tebbi A., Han Y.H., O’Neill B.T., Cooksey R.C., Jones D., Holland W.L., McClain D.A. (2012). Early mitochondrial adaptations in skeletal muscle to diet-induced obesity are strain dependent and determine oxidative stress and energy expenditure but not insulin sensitivity. Endocrinology.

[10] Tang Z., Wang P., Dong C., Zhang J., Wang X., Pei H. (2022). Oxidative Stress Signaling Mediated Pathogenesis of Diabetic Cardiomyopathy. Oxid. Med. Cell Longev..

[11] Kaludercic N., Di Lisa F. (2020). Mitochondrial ROS Formation in the Pathogenesis of Diabetic Cardiomyopathy. Front. Cardiovasc. Med..

[12] Turner N.A., Mughal R.S., Warburton P., O’Regan D.J., Ball S.G., Porter K.E. (2007). Mechanism of TNFalpha-induced IL-1alpha, IL-1beta and IL-6 expression in human cardiac fibroblasts: Effects of statins and thiazolidinediones. Cardiovasc. Res..

[13] Kadoglou N.P., Sailer N., Moumtzouoglou A., Kapelouzou A., Gerasimidis T., Kostakis A., Liapis C.D. (2012). Adipokines: A novel link between adiposity and carotid plaque vulnerability. Eur. J. Clin. Investig..

[14] Mengstie M.A., Abebe E.C., Teklemariam A.B., Mulu A.T., Teshome A.A., Zewde E.A., Muche Z.T., Azezew M.T. (2022). Molecular and cellular mechanisms in diabetic heart failure: Potential therapeutic targets. Front. Endocrinol..

[15] Dhar A., Venkadakrishnan J., Roy U., Vedam S., Lalwani N., Ramos K.S., Pandita T.K., Bhat A. (2023). A comprehensive review of the novel therapeutic targets for the treatment of diabetic cardiomyopathy. Ther. Adv. Cardiovasc. Dis..

[16] Ke J., Pan J., Lin H., Gu J. (2023). Diabetic cardiomyopathy: A brief summary on lipid toxicity. ESC Heart Fail..

[17] Ma X.M., Geng K., Law B.Y., Wang P., Pu Y.L., Chen Q., Xu H.W., Tan X.Z., Jiang Z.Z., Xu Y. (2023). Lipotoxicity-induced mtDNA release promotes diabetic cardiomyopathy by activating the cGAS-STING pathway in obesity-related diabetes. Cell Biol. Toxicol..

[18] Wang M., Li Y., Li S., Lv J. (2022). Endothelial Dysfunction and Diabetic Cardiomyopathy. Front. Endocrinol..

[19] Sena C.M., Pereira A.M., Seiça R. (2013). Endothelial dysfunction—A major mediator of diabetic vascular disease. Biochim. Biophys. Acta.

[20] Dhalla N.S., Shah A.K., Tappia P.S. (2020). Role of Oxidative Stress in Metabolic and Subcellular Abnormalities in Diabetic Cardiomyopathy. Int. J. Mol. Sci..

[21] Arad M., Waldman M., Abraham N.G., Hochhauser E. (2020). Therapeutic approaches to diabetic cardiomyopathy: Targeting the antioxidant pathway. Prostaglandins Other Lipid Mediat..

[22] Batista J.P.T., Faria A.O.V., Ribeiro T.F.S., Simões ESilva A.C. (2023). The Role of Renin-Angiotensin System in Diabetic Cardiomyopathy: A Narrative Review. Life.

[23] Quinaglia T., Oliveira D.C., Matos-Souza J.R., Sposito A.C. (2019). Diabetic cardiomyopathy: Factual or factoid?. Rev. Assoc. Med. Bras. (1992).

[24] Brown N.J. (2013). Contribution of aldosterone to cardiovascular and renal inflammation and fibrosis. Nat. Rev. Nephrol..

[25] Li J., Xie Y., Zheng S., He H., Wang Z., Li X., Jiao S., Liu D., Yang F., Zhao H. (2024). Targeting autophagy in diabetic cardiomyopathy: From molecular mechanisms to pharmacotherapy. Biomed. Pharmacother..

[26] Zheng H., Zhu H., Liu X., Huang X., Huang A., Huang Y. (2021). Mitophagy in Diabetic Cardiomyopathy: Roles and Mechanisms. Front. Cell Dev. Biol..

[27] Jia G., Hill M.A., Sowers J.R. (2018). Diabetic Cardiomyopathy: An Update of Mechanisms Contributing to This Clinical Entity. Circ. Res..

[28] Al Kury L.T. (2020). Calcium Homeostasis in Ventricular Myocytes of Diabetic Cardiomyopathy. J. Diabetes Res..

[29] Zhou Q., Lv D., Chen P., Xu T., Fu S., Li J., Bei Y. (2014). MicroRNAs in diabetic cardiomyopathy and clinical perspectives. Front. Genet..

[30] Li H., Fan J., Chen C., Wang D.W. (2020). Subcellular microRNAs in diabetic cardiomyopathy. Ann. Transl. Med..

[31] Paolillo S., Marsico F., Prastaro M., Renga F., Esposito L., De Martino F., Di Napoli P., Esposito I., Ambrosio A., Ianniruberto M. (2019). Diabetic cardiomyopathy: Definition, diagnosis, and therapeutic implications. Heart Fail. Clin..

[32] Seferović P.M., Paulus W.J. (2015). Clinical diabetic cardiomyopathy: A two-faced disease with restrictive and dilated phenotypes. Eur. Heart J..

[33] Zhao X., Liu S., Wang X., Chen Y., Pang P., Yang Q., Lin J., Deng S., Wu S., Fan G. (2022). Diabetic cardiomyopathy: Clinical phenotype and practice. Front. Endocrinol..

[34] Shou Y., Li X., Fang Q., Xie A., Zhang Y., Fu X., Wang M., Gong W., Zhang X., Yang D. (2024). Progress in the treatment of diabetic cardiomyopathy, a systematic review. Pharmacol. Res. Perspect..

[35] Nakamura M., Sadoshima J. (2020). Cardiomyopathy in obesity, insulin resistance and diabetes. J. Physiol..

[36] Ren J., Wu N.N., Wang S., Sowers J.R., Zhang Y. (2021). Obesity cardiomyopathy: Evidence, mechanisms, and therapeutic implications. Physiol. Rev..

[37] Salvatore T., Pafundi P.C., Galiero R., Albanese G., Di Martino A., Caturano A., Vetrano E., Rinaldi L., Sasso F.C. (2021). The Diabetic Cardiomyopathy: The Contributing Pathophysiological Mechanisms. Front. Med..

[38] Nakamura K., Miyoshi T., Yoshida M., Akagi S., Saito Y., Ejiri K., Matsuo N., Ichikawa K., Iwasaki K., Naito T. (2022). Pathophysiology and Treatment of Diabetic Cardiomyopathy and Heart Failure in Patients with Diabetes Mellitus. Int. J. Mol. Sci..

[39] Rena G., Hardie D.G., Pearson E.R. (2017). The mechanisms of action of metformin. Diabetologia.

[40] Eurich D.T., Weir D.L., Majumdar S.R., Tsuyuki R.T., Johnson J.A., Tjosvold L., Vanderloo S.E., McAlister F.A. (2013). Comparative safety and effectiveness of metformin in patients with diabetes mellitus and heart failure: Systematic review of observational studies involving 34,000 patients. Circ. Heart Fail..

[41] Marino A., Hausenloy D.J., Andreadou I., Horman S., Bertrand L., Beauloye C. (2021). AMP-activated protein kinase: A remarkable contributor to preserve a healthy heart against ROS injury. Free Radic. Biol. Med..

[42] Zhou G., Myers R., Li Y., Chen Y., Shen X., Fenyk-Melody J., Wu M., Ventre J., Doebber T., Fujii N. (2001). Role of AMP-activated protein kinase in mechanism of metformin action. J. Clin. Investig..

[43] Bu Y., Peng M., Tang X., Xu X., Wu Y., Chen A.F., Yang X. (2022). Protective effects of metformin in various cardiovascular diseases: Clinical evidence and AMPK-dependent mechanisms. J. Cell. Mol. Med..

[44] Salvatore T., Galiero R., Caturano A., Vetrano E., Rinaldi L., Coviello F., Di Martino A., Albanese G., Marfella R., Sardu C. (2021). Effects of Metformin in Heart Failure: From Pathophysiological Rationale to Clinical Evidence. Biomolecules.

[45] Salvatore T., Galiero R., Caturano A., Vetrano E., Rinaldi L., Coviello F., Di Martino A., Albanese G., Marfella R., Sardu C. (2009). Activation of AMP-activated protein kinase by metformin improves left ventricular function and survival in heart failure. Circ. Res..

[46] Kamel A.M., Sabry N., Farid S. (2022). Effect of metformin on left ventricular mass and functional parameters in non-diabetic patients: A meta-analysis of randomized clinical trials. BMC Cardiovasc. Disord..

[47] Kadoglou N.P., Kapelouzou A., Tsanikidis H., Vitta I., Liapis C.D., Sailer N. (2011). Effects of rosiglitazone/metformin fixed-dose combination therapy and metformin monotherapy on serum vaspin, adiponectin and IL-6 levels in drug-naïve patients with type 2 diabetes. Exp. Clin. Endocrinol. Diabetes.

[48] Crowley M.J., Diamantidis C.J., McDuffie J.R., Cameron C.B., Stanifer J.W., Mock C.K., Wang X., Tang S., Nagi A., Kosinski A.S. (2017). Clinical Outcomes of Metformin Use in Populations with Chronic Kidney Disease, Congestive Heart Failure, or Chronic Liver Disease: A Systematic Review. Ann. Intern. Med..

[49] Mavrogeni S.I., Bacopoulou F., Markousis-Mavrogenis G., Giannakopoulou A., Kariki O., Vartela V., Kolovou G., Charmandari E., Chrousos G. (2021). Cardiovascular Magnetic Resonance as Pathophysiologic Tool in Diabetes Mellitus. Front. Endocrinol..

[50] Anson M., Zhao S.S., Essa H., Austin P., Ibarburu G.H., Lip G.Y.H., Alam U. (2023). Metformin and SGLT2i as First-line Combination Therapy in Type 2 Diabetes: A Real-world Study with a Focus on Ethnicity. Clin. Ther..

[51] van Baar M.J.B., van Ruiten C.C., Muskiet M.H.A., van Bloemendaal L., IJzerman R.G., van Raalte D.H. (2018). SGLT2 Inhibitors in Combination Therapy: From Mechanisms to Clinical Considerations in Type 2 Diabetes Management. Diabetes Care.

[52] Karamanou M., Protogerou A., Tsoucalas G., Androutsos G., Poulakou-Rebelakou E. (2016). Milestones in the history of diabetes mellitus: The main contributors. World J. Diabetes.

[53] DiNicolantonio J.J., O’Keefe J.H. (2022). Added Sugars Drive Insulin Resistance, Hyperinsulinemia, Hypertension, Type 2 Diabetes and Coronary Heart Disease. Mo. Med..

[54] Cong S., Ramachandra C.J.A., Mai Ja K.M., Yap J., Shim W., Wei L., Hausenloy D.J. (2020). Mechanisms underlying diabetic cardiomyopathy: From pathophysiology to novel therapeutic targets. Cond. Med..

[55] Daoud Naccache D., Yalonetsky S., Bar-Yoseph R. (2020). Acute Effects of Insulin on Cardiac Function in Patients with Diabetes Mellitus: Clinical Applicability and Feasibility. Int. J. Endocrinol..

[56] Klein L.J., Visser F.C. (2010). The effect of insulin on the heart: Part 1: Effects on metabolism and function. Neth. Heart J..

[57] Negishi K. (2018). Echocardiographic feature of diabetic cardiomyopathy: Where are we now?. Cardiovasc. Diagn. Ther..

[58] Herman M.E., O’Keefe J.H., Bell D.S.H., Schwartz S.S. (2017). Insulin Therapy Increases Cardiovascular Risk in Type 2 Diabetes. Prog. Cardiovasc. Dis..

[59] Ahmad A., Sabbour H. (2024). Correction to: Effectiveness and safety of the combination of sodium-glucose transport protein 2 inhibitors and glucagon-like peptide-1 receptor agonists in patients with type 2 diabetes mellitus: A systematic review and meta-analysis of observational studies. Cardiovasc. Diabetol..

[60] Du S., Shi H., Xiong L., Wang P., Shi Y. (2022). Canagliflozin mitigates ferroptosis and improves myocardial oxidative stress in mice with diabetic cardiomyopathy. Front. Endocrinol..

[61] Arow M., Waldman M., Yadin D., Nudelman V., Shainberg A., Abraham N.G., Freimark D., Kornowski R., Aravot D., Hochhauser E. (2020). Sodium-glucose cotransporter 2 inhibitor Dapagliflozin attenuates diabetic cardiomyopathy. Cardiovasc. Diabetol..

[62] Al-Shamasi A.A., Elkaffash R., Mohamed M., Rayan M., Al-Khater D., Gadeau A.P., Ahmed R., Hasan A., Eldassouki H., Yalcin H.C. (2021). Crosstalk between Sodium-Glucose Cotransporter Inhibitors and Sodium-Hydrogen Exchanger 1 and 3 in Cardiometabolic Diseases. Int. J. Mol. Sci..

[63] He Q., Harding P., LaPointe M.C. (2010). PKA, Rap1, ERK1/2, and p90RSK mediate PGE2 and EP4 signaling in neonatal ventricular myocytes. Am. J. Physiol. Heart Circ. Physiol..

[64] Vallés P.G., Bocanegra V., Gil Lorenzo A., Costantino V.V. (2015). Physiological Functions and Regulation of the Na^+^/H^+^ Exchanger [NHE1] in Renal Tubule Epithelial Cells. Kidney Blood Press. Res..

[65] Wang D.D., Naumova A.V., Isquith D., Sapp J., Huynh K.A., Tucker I., Balu N., Voronyuk A., Chu B., Ordovas K. (2024). Dapagliflozin Reduces Systemic Inflammation in Patients with Type 2 Diabetes Without Known Heart Failure. Res. Sq..

[66] Theofilis P., Sagris M., Oikonomou E., Antonopoulos A.S., Siasos G., Tsioufis K., Tousoulis D. (2022). The impact of SGLT2 inhibitors on inflammation: A systematic review and meta-analysis of studies in rodents. Int. Immunopharmacol..

[67] Neal B., Perkovic V., Matthews D.R. (2017). Canagliflozin and Cardiovascular and Renal Events in Type 2 Diabetes. N. Engl. J. Med..

[68] Zinman B., Wanner C., Lachin J.M., Fitchett D., Bluhmki E., Hantel S., Mattheus M., Devins T., Johansen O.E., Woerle H.J. (2015). Empagliflozin, Cardiovascular Outcomes, and Mortality in Type 2 Diabetes. N. Engl. J. Med..

[69] Santos-Gallego C.G., Requena-Ibanez J.A., San Antonio R., Garcia-Ropero A., Ishikawa K., Watanabe S., Picatoste B., Vargas-Delgado A.P., Flores-Umanzor E.J., Sanz J. (2021). Empagliflozin Ameliorates Diastolic Dysfunction and Left Ventricular Fibrosis/Stiffness in Nondiabetic Heart Failure: A Multimodality Study. JACC Cardiovasc. Imaging.

[70] Brown A.J.M., Gandy S., McCrimmon R., Houston J.G., Struthers A.D., Lang C.C. (2020). A randomized controlled trial of dapagliflozin on left ventricular hypertrophy in people with type two diabetes: The DAPA-LVH trial. Eur. Heart J..

[71] Patel R., Wadid M., Makwana B., Kumar A., Khadke S., Bhatti A., Banker A., Husami Z., Labib S., Venesy D. (2024). GLP-1 Receptor Agonists Among Patients with Overweight or Obesity, Diabetes, and HFpEF on SGLT2 Inhibitors. JACC Heart Fail..

[72] Scisciola L., Chianese U., Caponigro V., Basilicata M.G., Salviati E., Altucci L., Campiglia P., Paolisso G., Barbieri M., Benedetti R. (2023). Multi-omics analysis reveals attenuation of cellular stress by empagliflozin in high glucose-treated human cardiomyocytes. J. Transl. Med..

[73] Li C., Zhang J., Xue M., Li X., Han F., Liu X., Xu L., Lu Y., Cheng Y., Li T. (2019). SGLT2 inhibition with empagliflozin attenuates myocardial oxidative stress and fibrosis in diabetic mice heart. Cardiovasc. Diabetol..

[74] Li N., Zhu Q.X., Li G.Z., Wang T., Zhou H. (2023). Empagliflozin ameliorates diabetic cardiomyopathy probably via activating AMPK/PGC-1alpha and inhibiting the RhoA/ROCK pathway. World J. Diabetes.

[75] Kadosaka T., Watanabe M., Natsui H., Koizumi T., Nakao M., Koya T., Hagiwara H., Kamada R., Temma T., Karube F. (2023). Empagliflozin attenuates arrhythmogenesis in diabetic cardiomyopathy by normalizing intracellular Ca(2+) handling in ventricular cardiomyocytes. Am. J. Physiol. Heart Circ. Physiol..

[76] Lo C.W.H., Fei Y., Cheung B.M.Y. (2021). Cardiovascular Outcomes in Trials of New Antidiabetic Drug Classes. Card. Fail. Rev..

[77] Saini K., Sharma S., Khan Y. (2023). DPP-4 inhibitors for treating T2DM—Hype or hope? An analysis based on the current literature. Front. Mol. Biosci..

[78] Udell J.A., Bhatt D.L., Braunwald E., Cavender M.A., Mosenzon O., Steg P.G., Davidson J.A., Nicolau J.C., Corbalan R., Hirshberg B. (2015). Saxagliptin and cardiovascular outcomes in patients with type 2 diabetes and moderate or severe renal impairment: Observations from the SAVOR-TIMI 53 Trial. Diabetes Care.

[79] Sandhu P., Ong J.P., Garg V., Altaha M., Bello O., Singal S.R., Verma S., Yan A.T., Connelly K.A. (2021). The effects of saxagliptin on cardiac structure and function using cardiac MRI (SCARF). Acta Diabetol..

[80] Inzucchi S.E., Bergenstal R.M., Buse J.B., Diamant M., Ferrannini E., Nauck M., Peters A.L., Tsapas A., Wender R., Matthews D.R. (2012). Management of hyperglycemia in type 2 diabetes: A patient-centered approach: Position statement of the American Diabetes Association (ADA) and the European Association for the Study of Diabetes (EASD). Diabetes Care.

[81] Patoulias D.I., Boulmpou A., Teperikidis E., Katsimardou A., Siskos F., Doumas M., Papadopoulos C.E., Vassilikos V. (2021). Cardiovascular efficacy and safety of dipeptidyl peptidase-4 inhibitors: A meta-analysis of cardiovascular outcome trials. World J. Cardiol..

[82] Müller T.D., Finan B., Bloom S.R., D’Alessio D., Drucker D.J., Flatt P.R., Fritsche A., Gribble F., Grill H.J., Habener J.F. (2019). Glucagon-like peptide 1 (GLP-1). Mol. Metab..

[83] Gerstein H.C., Colhoun H.M., Dagenais G.R., Diaz R., Lakshmanan M., Pais P., Probstfield J., Riesmeyer J.S., Riddle M.C., Rydén L. (2019). Dulaglutide and cardiovascular outcomes in type 2 diabetes (REWIND): A double-blind, randomised placebo-controlled trial. Lancet.

[84] Drucker D.J. (2016). The Cardiovascular Biology of Glucagon-like Peptide-1. Cell Metab..

[85] Bizino M.B., Jazet I.M., Westenberg J.J.M., van Eyk H.J., Paiman E.H.M., Smit J.W.A., Lamb H.J. (2019). Effect of liraglutide on cardiac function in patients with type 2 diabetes mellitus: Randomized placebo-controlled trial. Cardiovasc. Diabetol..

[86] Ida S., Kaneko R., Imataka K., Okubo K., Shirakura Y., Azuma K., Fujiwara R., Takahashi H., Murata K. (2021). Effects of oral antidiabetic drugs and glucagon-like peptide-1 receptor agonists on left ventricular diastolic function in patients with type 2 diabetes mellitus: A systematic review and network meta-analysis. Heart Fail. Rev..

[87] Marso S.P., Bain S.C., Consoli A., Eliaschewitz F.G., Jódar E., Leiter L.A., Lingvay I., Rosenstock J., Seufert J., Warren M.L. (2016). Semaglutide and Cardiovascular Outcomes in Patients with Type 2 Diabetes. N. Engl. J. Med..

[88] Huixing L., Di F., Daoquan P. (2023). Effect of Glucagon-like Peptide-1 Receptor Agonists on Prognosis of Heart Failure and Cardiac Function: A Systematic Review and Meta-analysis of Randomized Controlled Trials. Clin. Ther..

[89] Rivera F.B., Cruz L.L.A., Magalong J.V., Ruyeras J.M.M.J., Aparece J.P., Bantayan N.R.B., Lara-Breitinger K., Gulati M. (2024). Cardiovascular and renal outcomes of glucagon-like peptide 1 receptor agonists among patients with and without type 2 diabetes mellitus: A meta-analysis of randomized placebo-controlled trials. Am. J. Prev. Cardiol..

[90] Dunlay S.M., Givertz M.M., Aguilar D., Allen L.A., Chan M., Desai A.S., Deswal A., Dickson V.V., Kosiborod M.N., Lekavich C.L. (2019). Type 2 Diabetes Mellitus and Heart Failure: A Scientific Statement From the American Heart Association and the Heart Failure Society of America: This statement does not represent an update of the 2017 ACC/AHA/HFSA heart failure guideline update. Circulation.

[91] Fisman E.Z., Tenenbaum A. (2021). The dual glucose-dependent insulinotropic polypeptide (GIP) and glucagon-like peptide-1 (GLP-1) receptor agonist tirzepatide: A novel cardiometabolic therapeutic prospect. Cardiovasc. Diabetol..

[92] Giugliano D., Longo M., Signoriello S., Maiorino M.I., Solerte B., Chiodini P., Esposito K. (2022). The effect of DPP-4 inhibitors, GLP-1 receptor agonists and SGLT-2 inhibitors on cardiorenal outcomes: A network meta-analysis of 23 CVOTs. Cardiovasc. Diabetol..

[93] Basile P., Guaricci A.I., Piazzolla G., Volpe S., Vozza A., Benedetto M., Carella M.C., Santoro D., Monitillo F., Baggiano A. (2023). Improvement of Left Ventricular Global Longitudinal Strain after 6-Month Therapy with GLP-1RAs Semaglutide and Dulaglutide in Type 2 Diabetes Mellitus: A Pilot Study. J. Clin. Med..

[94] Pan K.L., Hsu Y.C., Chang S.T., Chung C.M., Lin C.L. (2023). The Role of Cardiac Fibrosis in Diabetic Cardiomyopathy: From Pathophysiology to Clinical Diagnostic Tools. Int. J. Mol. Sci..

[95] Kadoglou N.P., Vrabas I.S., Kapelouzou A., Angelopoulou N. (2012). The association of physical activity with novel adipokines in patients with type 2 diabetes. Eur. J. Intern. Med..

[96] Kristensen S.L., Rørth R., Jhund P.S., Docherty K.F., Sattar N., Preiss D., Køber L., Petrie M.C., McMurray J.J.V. (2019). Cardiovascular, mortality, and kidney outcomes with GLP-1 receptor agonists in patients with type 2 diabetes: A systematic review and meta-analysis of cardiovascular outcome trials. Lancet Diabetes Endocrinol..

[97] Montaigne D., Butruille L., Staels B. (2021). PPAR control of metabolism and cardiovascular functions. Nat. Rev. Cardiol..

[98] Nikolaidis L.A., Levine T.B. (2004). Peroxisome proliferator activator receptors (PPAR), insulin resistance, and cardiomyopathy: Friends or foes for the diabetic patient with heart failure?. Cardiol. Rev..

[99] Zheng J., Cheng J., Zheng S., Zhang L., Guo X., Zhang J., Xiao X. (2018). Physical Exercise and Its Protective Effects on Diabetic Cardiomyopathy: What Is the Evidence?. Front. Endocrinol..

[100] Garvey W.T., Hardin D., Juhaszova M., Dominguez J.H. (1993). Effects of diabetes on myocardial glucose transport system in rats: Implications for diabetic cardiomyopathy. Am. J. Physiol..

[101] Waller A.P., Kalyanasundaram A., Hayes S., Periasamy M., Lacombe V.A. (2015). Sarcoplasmic reticulum Ca^2+^ ATPase pump is a major regulator of glucose transport in the healthy and diabetic heart. Biochim. Biophys. Acta.

[102] Murray J., Bennett H., Bezak E., Perry R., Boyle T. (2022). The effect of exercise on left ventricular global longitudinal strain. Eur. J. Appl. Physiol..

[103] Wang H., Bei Y., Lu Y., Sun W., Liu Q., Wang Y., Cao Y., Chen P., Xiao J., Kong X. (2015). Exercise Prevents Cardiac Injury and Improves Mitochondrial Biogenesis in Advanced Diabetic Cardiomyopathy with PGC-1alpha and Akt Activation. Cell Physiol. Biochem..

[104] Jung I. (2022). Changes in Patterns of Physical Activity and Risk of Heart Failure in Newly Diagnosed Diabetes Mellitus Patients. Diabetes Metab. J..

[105] Liu X. (2022). Association between physical exercise and all-cause and CVD mortality in patients with diabetes: An updated systematic review and meta-analysis. Afr. Health Sci..

[106] Karjalainen J.J., Kiviniemi A.M., Hautala A.J., Piira O.P., Lepojärvi E.S., Perkiömäki J.S., Junttila M.J., Huikuri H.V., Tulppo M.P. (2015). Effects of physical activity and exercise training on cardiovascular risk in coronary artery disease patients with and without type 2 diabetes. Diabetes Care.

[107] Colberg S.R., Sigal R.J., Yardley J.E., Riddell M.C., Dunstan D.W., Dempsey P.C., Horton E.S., Castorino K., Tate D.F. (2016). Physical Activity/Exercise and Diabetes: A Position Statement of the American Diabetes Association. Diabetes Care.

[108] Kenny H.C., Abel E.D. (2019). Heart failure in type 2 diabetes mellitus. Circ. Res..

[109] Mascolo A., di Mauro G., Cappetta D., De Angelis A., Torella D., Urbanek K., Berrino L., Nicoletti G.F., Capuano A., Rossi F. (2022). Current and future therapeutic perspective in chronic heart failure. Pharmacol. Res..

[110] Rösen R., Rump A.F., Rösen P. (1995). The ACE-inhibitor captopril improves myocardial perfusion in spontaneously diabetic (BB) rats. Diabetologia.

[111] Yaras N., Bilginoglu A., Vassort G., Turan B. (2007). Restoration of diabetes induced abnormal local Ca^2+^ release in cardiomyocytes by angiotensin II receptor blockade. Am. J. Physiol. Heart Circ. Physiol..

[112] Rydén L., Armstrong P.W., Cleland J.G.F., Horowitz J.D., Massie B.M., Packer M., Poole-Wilson P.A. (2000). Efficacy and safety of high-dose lisinopril in chronic heart failure patients at high cardiovascular risk, including those with diabetes mellitus. Results from the ATLAS trial. Eur. Heart J..

[113] Kadoglou N.P.E., Parissis J., Karavidas A., Kanonidis I., Trivella M. (2022). Assessment of acute heart failure prognosis: The promising role of prognostic models and biomarkers. Heart Fail. Rev..

[114] Sleight P. (2000). The HOPE Study (Heart Outcomes Prevention Evaluation). J. Renin Angiotensin Aldosterone Syst..

[115] Young J.B., Dunlap M.E., Pfeffer M.A., Probstfield J.L., Cohen-Solal A., Dietz R., Granger C.B., Hradec J., Kuch J., McKelvie R.S. (2004). Mortality and morbidity reduction with Candesartan in patients with chronic heart failure and left ventricular systolic dysfunction: Results of the CHARM low-left ventricular ejection fraction trials. Circulation.

[116] Brenner B.M., Cooper M.E., de Zeeuw D., Keane W.F., Mitch W.E., Parving H.H., Remuzzi G., Snapinn S.M., Zhang Z., Shahinfar S. (2001). Effects of losartan on renal and cardiovascular outcomes in patients with type 2 diabetes and nephropathy. N. Engl. J. Med..

[117] Leoncini G., Viazzi F., De Cosmo S., Russo G., Fioretto P., Pontremoli R. (2020). Blood pressure reduction and RAAS inhibition in diabetic kidney disease: Therapeutic potentials and limitations. J. Nephrol..

[118] Gillespie E.L., White C.M., Kardas M., Lindberg M., Coleman C.I. (2005). The impact of ACE inhibitors or angiotensin II type 1 receptor blockers on the development of new-onset type 2 diabetes. Diabetes Care.

[119] Segura J., Ruilope L.M. (2007). Obesity, essential hypertension and renin-angiotensin system. Public Health Nutr..

[120] Dzau V.J., Colucci W.S., Williams G.H., Curfman G., Meggs L., Hollenberg N.K. (1980). Sustained effectiveness of converting-enzyme inhibition in patients with severe congestive heart failure. N. Engl. J. Med..

[121] Beldhuis I.E., Lam C.S.P., Testani J.M., Voors A.A., Van Spall H.G.C., Ter Maaten J.M., Damman K. (2022). Evidence-based medical therapy in patients with heart failure with reduced ejection fraction and chronic kidney disease. Circulation.

[122] Ge Q., Zhao L., Ren X.M., Ye P., Hu Z.Y. (2019). LCZ696, an angiotensin receptor-neprilysin inhibitor, ameliorates diabetic cardiomyopathy by inhibiting inflammation, oxidative stress and apoptosis. Exp. Biol. Med..

[123] Wu W., Shi F., Liu D., Ceddia R.P., Gaffin R., Wei W., Fang H., Lewandowski E.D., Collins S. (2017). Enhancing natriuretic peptide signaling in adipose tissue, but not in muscle, protects against diet-induced obesity and insulin resist ance. Sci. Signal..

[124] Seferovic J.P., Claggett B., Seidelmann S.B., Seely E.W., Packer M., Zile M.R., Rouleau J.L., Swedberg K., Lefkowitz M., Shi V.C. (2017). Effect of Sacubitril/Valsartan versus enalapril on glycaemic control in patients with heart failure and diabetes: A post-hoc analysis from the paradigm-hf trial. Lancet Diabetes Endocrinol..

[125] Wang R., Ye H., Zhao Y., Wei J., Wang Y., Zhang X., Wang L. (2022). Effect of sacubitril/valsartan and ACEI/ARB on glycaemia and the development of diabetes: A systematic review and meta-analysis of randomised controlled trials. BMC Med..

[126] Kim H.M., Hwang I.C., Choi W., Yoon Y.E., Cho G.Y. (2021). Combined effects of ARNI and SGLT2 inhibitors in diabetic patients with heart failure with reduced ejection fraction. Sci. Rep..

[127] Zhang X., Zhou Y., Ma R. (2022). Potential effects and application prospect of angiotensin receptor-neprilysin inhibitor in diabetic kidney disease. J. Diabetes Complicat..

[128] Liu W., Gong W., He M., Liu Y., Yang Y., Wang M., Wu M., Guo S., Yu Y., Wang X. (2018). Spironolactone Protects against Diabetic Cardiomyopathy in Streptozotocin-Induced Diabetic Rats. J. Diabetes Res..

[129] Ramírez E., Klett-Mingo M., Ares-Carrasco S., Picatoste B., Ferrarini A., Rupérez F.J., Caro-Vadillo A., Barbas C., Egido J., Tuñón J. (2013). Eplerenone attenuated cardiac steatosis, apoptosis and diastolic dysfunction in experimental type-II diabetes. Cardiovasc. Diabetol..

[130] Ferreira J.P., Cleland J.G., Girerd N., Bozec E., Rossignol P., Pellicori P., Cosmi F., Mariottoni B., Solomon S.D., Pitt B. (2023). Spironolactone effect on cardiac structure and function of patients with heart failure and preserved ejection fraction: A pooled analysis of three randomized trials. Eur. J. Heart Fail..

[131] Chen M.D., Dong S.S., Cai N.Y., Fan M.D., Gu S.P., Zheng J.J., Yin H.M., Zhou X.H., Wang L.X., Li C.Y. (2016). Efficacy and safety of mineralocorticoid receptor antagonists for patients with heart failure and diabetes mellitus: A systematic review and meta-analysis. BMC Cardiovasc. Disord..

[132] Chen X., Huang M., Chen Y., Xu H., Wu M. (2025). Mineralocorticoid receptor antagonists and heart failure with preserved ejection fraction: Current understanding and future prospects. Heart Fail. Rev..

[133] Jin T., Fu X., Liu M., An F. (2023). Finerenone attenuates myocardial apoptosis, metabolic disturbance and myocardial fibrosis in type 2 diabetes mellitus. Diabetol. Metab. Syndr..

[134] Agarwal R., Filippatos G., Pitt B., Anker S.D., Rossing P., Joseph A., Kolkhof P., Nowack C., Gebel M., Ruilope L.M. (2022). Cardiovascular and kidney outcomes with finerenone in patients with type 2 diabetes and chronic kidney disease: The FIDELITY pooled analysis. Eur. Heart J..

[135] Wang J., Xue H., He J., Deng L., Tian J., Jiang Y., Feng J. (2024). Therapeutic potential of finerenone for diabetic cardiomyopathy: Focus on the mechanisms. Diabetol. Metab. Syndr..

[136] Thackeray J.T., Beanlands R.S., Dasilva J.N. (2012). Altered sympathetic nervous system signaling in the diabetic heart: Emerging targets for molecular imaging. Am. J. Nucl. Med. Mol. Imaging.

[137] David S.H.B. (2004). Advantages of a third-generation β-blocker in patients with diabetes mellitus. Am. J. Cardiol..

[138] Haas S.J., Vos T., Gilbert R.E., Krum H. (2003). Are beta-blockers as efficacious in patients with diabetes mellitus as in patients without diabetes mellitus who have chronic heart failure? A meta-analysis of large-scale clinical trials. Am. Heart J..

[139] Silverman D.N., Plante T.B., Infeld M., Callas P.W., Juraschek S.P., Dougherty G.B., Meyer M. (2019). Association of β-Blocker Use with Heart Failure Hospitalizations and Cardiovascular Disease Mortality Among Patients with Heart Failure with a Preserved Ejection Fraction: A Secondary Analysis of the TOPCAT Trial. JAMA Netw. Open.

[140] Palau P., Seller J., Domínguez E., Sastre C., Ramón J.M., de La Espriella R., Santas E., Miñana G., Bodí V., Sanchis J. (2021). Effect of β-blocker withdrawal on functional capacity in heart failure and preserved ejection fraction. J. Am. Coll. Cardiol..

[141] Peikert A., Bart B.A., Vaduganathan M., Claggett B.L., Kulac I.J., Kosiborod M.N., Desai A.S., Jhund P.S., Lam C.S.P., Inzucchi S.E. (2024). Contemporary Use and Implications of Beta-Blockers in Patients with HFmrEF or HFpEF: The DELIVER Trial. J. Am. Coll. Cardiol. Heart Fail..

[142] Nakamura K., Kusano K., Nakamura Y., Kakishita M., Ohta K., Nagase S., Yamamoto M., Miyaji K., Saito H., Morita H. (2002). Carvedilol decreases elevated oxidative stress in human failing myocardium. Circulation.

[143] Popp D.A., Tse T.F., Shah S.D., Clutter W.E., Cryer P.E. (1984). Oral propranolol and metoprolol both impair glucose recovery from insulin-induced hypoglycemia in insulin-dependent diabetes mellitus. Diabetes Care.

[144] Weinberger M.H. (1985). Antihypertensive therapy and lipids: Evidence, mechanisms, and implications. Arch. Intern. Med..

[145] Felker G.M., Lee K.L., Bull D.A., Redfield M.M., Stevenson L.W., Goldsmith S.R., LeWinter M.M., Deswal A., Rouleau J.L., Ofili E.O. (2011). Diuretic strategies in patients with acute decompensated heart failure. N. Engl. J. Med..

[146] Chatur S., Vaduganathan M., Claggett B., Vardeny O., Desai A.S., Jhund P.S., de Boer R.A., Lam C.S.P., Kosiborod M.N., Shah S.J. (2023). Dapagliflozin and diuretic utilization in heart failure with mildly reduced or preserved ejection fraction: The DELIVER trial. Eur. Heart J..

[147] Kalinski J.K., Xu B., Boyd R., Tasseff N., Rutkowski K., Ospina S., Smedira N., Thamilarasan M., Popovic Z.B., Desai M.Y. (2024). Novel Cardiac Myosin Inhibitor Therapy for Hypertrophic Cardiomyopathy in Adults: A Contemporary Review. Am. J. Cardiovasc. Drugs.

[148] Braunwald E., Saberi S., Abraham T.P., Elliott P.M., Olivotto I. (2023). Mavacamten: A first-in-class myosin inhibitor for obstructive hypertrophic cardiomyopathy. Eur. Heart J..

[149] Sun X., Pang H., Li J., Luo S., Huang G., Li X., Xie Z., Zhou Z. (2020). The NLRP3 Inflammasome and Its Role in T1DM. Front. Immunol..

[150] Cao R., Fang D., Wang J., Yu Y., Ye H., Kang P., Li Z., Wang H., Gao Q. (2019). ALDH2 Overexpression Alleviates High Glucose-Induced Cardiotoxicity by Inhibiting NLRP3 Inflammasome Activation. J. Diabetes Res..

[151] Li X., Kerindongo R.P., Preckel B., Kalina J.O., Hollmann M.W., Zuurbier C.J., Weber N.C. (2023). Canagliflozin inhibits inflammasome activation in diabetic endothelial cells—Revealing a novel calcium-dependent anti-inflammatory effect of canagliflozin on human diabetic endothelial cells. Biomed. Pharmacother..

[152] Kadoglou N.P.E., Christodoulou E., Kostomitsopoulos N., Valsami G. (2021). The cardiovascular-protective properties of saffron and its potential pharmaceutical applications: A critical appraisal of the literature. Phytother. Res..

[153] Sun S., Yang S., An N., Wang G., Xu Q., Liu J., Mao Y. (2019). Astragalus polysaccharides inhibits cardiomyocyte apoptosis during diabetic cardiomyopathy via the endoplasmic reticulum stress pathway. J. Ethnopharmacol..

[154] Li L., Chen X., Su C., Wang Q., Li R., Jiao W., Luo H., Tian Y., Tang J., Li X. (2020). Si-Miao-Yong-An decoction preserves cardiac function and regulates GLC/AMPK/NF-κB and GLC/PPARα/PGC-1α pathways in diabetic mice. Biomed. Pharmacother..

[155] Wen H.L., Liang Z.S., Zhang R., Yang K. (2013). Anti-inflammatory effects of triptolide improve left ventricular function in a rat model of diabetic cardiomyopathy. Cardiovasc. Diabetol..

[156] Guo X., Xue M., Li C.J., Yang W., Wang S.S., Ma Z.J., Zhang X.N., Wang X.Y., Zhao R., Chang B.C. (2016). Protective effects of triptolide on TLR4 mediated autoimmune and inflammatory response induced myocardial fibrosis in diabetic cardiomyopathy. J. Ethnopharmacol..

[157] Zhang B., Zhang C.Y., Zhang X.L., Sun G.B., Sun X.B. (2021). Guan Xin Dan Shen formulation protects db/db mice against diabetic cardiomyopathy via activation of Nrf2 signaling. Mol. Med. Rep..

[158] Ni Q., Wang J., Li E.Q., Zhao A.B., Yu B., Wang M., Huang C.R. (2011). Study on the protective effect of shengmai san (see text) on the myocardium in the type 2 diabetic cardiomyopathy model rat. J. Tradit. Chin. Med..

[159] Tian J., Tang W., Xu M., Zhang C., Zhao P., Cao T., Shan X., Lu R., Guo W. (2018). Shengmai San Alleviates Diabetic Cardiomyopathy Through Improvement of Mitochondrial Lipid Metabolic Disorder. Cell Physiol. Biochem..

[160] Kim Y., Clifton P. (2018). Curcumin, Cardiometabolic Health and Dementia. Int. J. Environ. Res. Public Health.

[161] Soetikno V., Sari F.R., Sukumaran V., Lakshmanan A.P., Mito S., Harima M., Thandavarayan R.A., Suzuki K., Nagata M., Takagi R. (2012). Curcumin prevents diabetic cardiomyopathy in streptozotocin-induced diabetic rats: Possible involvement of PKC-MAPK signaling pathway. Eur. J. Pharm. Sci..

[162] Wei Z., Pinfang K., Jing Z., Zhuoya Y., Shaohuan Q., Chao S. (2023). Curcumin Improves Diabetic Cardiomyopathy by Inhibiting Pyroptosis through AKT/Nrf2/ARE Pathway. Mediat. Inflamm..

[163] Atale N., Yadav D., Rani V., Jin J.O. (2020). Pathophysiology, Clinical Characteristics of Diabetic Cardiomyopathy: Therapeutic Potential of Natural Polyphenols. Front. Nutr..

[164] Laddha A.P., Kulkarni Y.A. (2020). NADPH oxidase: A membrane-bound enzyme and its inhibitors in diabetic complications. Eur. J. Pharmacol..

[165] Januzzi J.L., Butler J., Del Prato S., Ezekowitz J.A., Ibrahim N.E., Lam C.S.P., Lewis G.D., Marwick T.H., Rosenstock J., Tang W.H.W. (2023). Rationale and design of the Aldose Reductase Inhibition for Stabilization of Exercise Capacity in Heart Failure Trial (ARISE-HF) in patients with high-risk diabetic cardiomyopathy. Am. Heart J..

[166] Gopal K., Karwi Q.G., Tabatabaei Dakhili S.A., Wagg C.S., Zhang L., Sun Q., Saed C.T., Panidarapu S., Perfetti R., Ramasamy R. (2023). Aldose reductase inhibition alleviates diabetic cardiomyopathy and is associated with a decrease in myocardial fatty acid oxidation. Cardiovasc. Diabetol..

[167] Kadoglou N.P.E., Papadopoulos C.H., Papadopoulos K.G., Karagiannis S., Karabinos I., Loizos S., Theodosis-Georgilas A., Aggeli K., Keramida K., Klettas D. (2022). Updated knowledge and practical implementations of stress echocardiography in ischemic and non-ischemic cardiac diseases: An expert consensus of the Working Group of Echocardiography of the Hellenic Society of Cardiology. Hell. J. Cardiol..

[168] Peng M.L., Fu Y., Wu C.W., Zhang Y., Ren H., Zhou S.S. (2022). Signaling Pathways Related to Oxidative Stress in Diabetic Cardiomyopathy. Front. Endocrinol..

[169] Huynh K., Bernardo B.C., McMullen J.R., Ritchie R.H. (2014). Diabetic cardiomyopathy: Mechanisms and new treatment strategies targeting antioxidant signaling pathways. Pharmacol. Ther..

[170] De Blasio M.J., Huynh K., Qin C., Rosli S., Kiriazis H., Ayer A., Cemerlang N., Stocker R., Du X.J., McMullen J.R. (2015). Therapeutic targeting of oxidative stress with coenzyme Q10 counteracts exaggerated diabetic cardiomyopathy in a mouse model of diabetes with diminished PI3K(p110α) signaling. Free Radic. Biol. Med..

[171] Jin B., Chen Y., Wang J., Chen Y., Zhang M., Huang J., Wang Y. (2023). Costunolide alleviates hyperglycaemia-induced diabetic cardiomyopathy via inhibiting inflammatory responses and oxidative stress. J. Cell Mol. Med..

[172] Li F., Hu Z., Huang Y., Zhan H. (2023). Dexmedetomidine ameliorates diabetic cardiomyopathy by inhibiting ferroptosis through the Nrf2/GPX4 pathway. J. Cardiothorac. Surg..

[173] Xie S.Y., Liu S.Q., Zhang T., Shi W.K., Xing Y., Fang W.X., Zhang M., Chen M.Y., Xu S.C., Fan M.Q. (2024). USP28 Serves as a Key Suppressor of Mitochondrial Morphofunctional Defects and Cardiac Dysfunction in the Diabetic Heart. Circulation.

[174] Yan M., Su L., Wu K., Mei Y., Liu Z., Chen Y., Zeng W., Xiao Y., Zhang J., Cai G. (2024). USP7 promotes cardiometabolic disorders and mitochondrial homeostasis dysfunction in diabetic mice via stabilizing PGC1β. Pharmacol. Res..

[175] Wang D., Yin Y., Wang S., Zhao T., Gong F., Zhao Y., Wang B., Huang Y., Cheng Z., Zhu G. (2021). FGF1ΔHBS prevents diabetic cardiomyopathy by maintaining mitochondrial homeostasis and reducing oxidative stress via AMPK/Nur77 suppression. Signal Transduct. Target. Ther..

[176] Guo P., Hu S., Liu X., He M., Li J., Ma T., Huang M., Fang Q., Wang Y. (2024). CAV3 alleviates diabetic cardiomyopathy via inhibiting NDUFA10-mediated mitochondrial dysfunction. J. Transl. Med..

[177] Wang H., Wang L., Hu F., Wang P., Xie Y., Li F., Guo B. (2022). Neuregulin-4 attenuates diabetic cardiomyopathy by regulating autophagy via the AMPK/mTOR signalling pathway. Cardiovasc. Diabetol..

[178] Hsuan C.F., Teng S.I.F., Hsu C.N., Liao D., Chang A.J., Lee H.L., Hee S.W., Chang Y.C., Chuang L.M. (2023). Emerging Therapy for Diabetic Cardiomyopathy: From Molecular Mechanism to Clinical Practice. Biomedicines.

[179] Varga Z.V., Giricz Z., Liaudet L., Haskó G., Ferdinandy P., Pacher P. (2015). Interplay of oxidative, nitrosative/nitrative stress, inflammation, cell death and autophagy in diabetic cardiomyopathy. Biochim. Biophys. Acta.

[180] Kanamori H., Naruse G., Yoshida A., Minatoguchi S., Watanabe T., Kawaguchi T., Yamada Y., Mikami A., Kawasaki M., Takemura G. (2019). Metformin Enhances Autophagy and Provides Cardioprotection in δ-Sarcoglycan Deficiency-Induced Dilated Cardiomyopathy. Circ. Heart Fail..

[181] Dewanjee S., Vallamkondu J., Kalra R.S., John A., Reddy P.H., Kandimalla R. (2021). Autophagy in the diabetic heart: A potential pharmacotherapeutic target in diabetic cardiomyopathy. Ageing Res. Rev..

[182] Sapian S., Taib I.S., Latip J., Katas H., Chin K.Y., Mohd Nor N.A., Jubaidi F.F., Budin S.B. (2021). Therapeutic Approach of Flavonoid in Ameliorating Diabetic Cardiomyopathy by Targeting Mitochondrial-Induced Oxidative Stress. Int. J. Mol. Sci..

[183] Jubaidi F.F., Zainalabidin S., Taib I.S., Hamid Z.A., Budin S.B. (2021). The Potential Role of Flavonoids in Ameliorating Diabetic Cardiomyopathy via Alleviation of Cardiac Oxidative Stress, Inflammation and Apoptosis. Int. J. Mol. Sci..

[184] Li L., Luo W., Qian Y., Zhu W., Qian J., Li J., Jin Y., Xu X., Liang G. (2019). Luteolin protects against diabetic cardiomyopathy by inhibiting NF-κB-mediated inflammation and activating the Nrf2-mediated antioxidant responses. Phytomedicine.

[185] Sun X., Chen R.C., Yang Z.H., Sun G.B., Wang M., Ma X.J., Yang L.J., Sun X.B. (2014). Taxifolin prevents diabetic cardiomyopathy in vivo and in vitro by inhibition of oxidative stress and cell apoptosis. Food Chem. Toxicol..

[186] Zhang B., Shen Q., Chen Y., Pan R., Kuang S., Liu G., Sun G., Sun X. (2017). Myricitrin Alleviates Oxidative Stress-induced Inflammation and Apoptosis and Protects Mice against Diabetic Cardiomyopathy. Sci. Rep..

[187] Qiao C., Wang H., Song Z., Ding Y., Tao J., Aa J., Ding X. (2020). Icariin Attenuates Diabetic Cardiomyopathy and Downregulates Extracellular Matrix Proteins in Heart Tissue of Type 2 Diabetic Rats. Pharmacology.

[188] Abukhalil M.H., Althunibat O.Y., Aladaileh S.H., Al-Amarat W., Obeidat H.M., Al-Khawalde A.A.A., Hussein O.E., Alfwuaires M.A., Algefare A.I., Alanazi K.M. (2021). Galangin attenuates diabetic cardiomyopathy through modulating oxidative stress, inflammation and apoptosis in rats. Biomed. Pharmacother..

[189] Uryash A., Mijares A., Flores V., Adams J.A., Lopez J.R. (2021). Effects of Naringin on Cardiomyocytes From a Rodent Model of Type 2 Diabetes. Front. Pharmacol..

[190] Abdel Rhman M., Owira P. (2022). The role of microRNAs in the pathophysiology, diagnosis, and treatment of diabetic cardiomyopathy. J. Pharm. Pharmacol..

[191] Kambis T.N., Mishra P.K. (2023). Genome Editing and Diabetic Cardiomyopathy. Adv. Exp. Med. Biol..

[192] Graczyk P., Dach A., Dyrka K., Pawlik A. (2024). Pathophysiology and Advances in the Therapy of Cardiomyopathy in Patients with Diabetes Mellitus. Int. J. Mol. Sci..

[193] Zhao S.F., Ye Y.X., Xu J.D., He Y., Zhang D.W., Xia Z.Y., Wang S. (2021). Long non-coding RNA KCNQ1OT1 increases the expression of PDCD4 by targeting miR-181a-5p, contributing to cardiomyocyte apoptosis in diabetic cardiomyopathy. Acta Diabetol..

[194] Wu Y., Leng Y., Meng Q., Xue R., Zhao B., Zhan L., Xia Z. (2017). Suppression of Excessive Histone Deacetylases Activity in Diabetic Hearts Attenuates Myocardial Ischemia/Reperfusion Injury via Mitochondria Apoptosis Pathway. J. Diabetes Res..

[195] Lee T.I., Kao Y.H., Tsai W.C., Chung C.C., Chen Y.C., Chen Y.J. (2016). HDAC Inhibition Modulates Cardiac PPARs and Fatty Acid Metabolism in Diabetic Cardiomyopathy. PPAR Res..

[196] Chen Y., Du J., Zhao Y.T., Zhang L., Lv G., Zhuang S., Qin G., Zhao T.C. (2015). Histone deacetylase (HDAC) inhibition improves myocardial function and prevents cardiac remodeling in diabetic mice. Cardiovasc. Diabetol..

